# Homozygous p.Ser267Phe in *SLC10A1* is associated with a new type of hypercholanemia and implications for personalized medicine

**DOI:** 10.1038/s41598-017-07012-2

**Published:** 2017-08-23

**Authors:** Ruihong Liu, Chuming Chen, Xuefeng Xia, Qijun Liao, Qiong Wang, Paul J. Newcombe, Shuhua Xu, Minghui Chen, Yue Ding, Xiaoying Li, Zhihong Liao, Fucheng Li, Minlian Du, Huaiqiu Huang, Ruimin Dong, Weiping Deng, Ye Wang, Binghui Zeng, Qihao Pan, Danhua Jiang, Hao Zeng, Pak Sham, Yingnan Cao, Patrick H. Maxwell, Zhi-liang Gao, Liang Peng, Yiming Wang

**Affiliations:** 1 Fifth Affiliated Hospital, Sun Yat-sen University-BGI Laboratory, Department of Experimental Medicine, The Fifth Affiliated Hospital,Sun Yat-sen University, Zhuhai, China; 2 Department of Infectious Diseases, Third Affiliated Hospital, Sun Yat-sen University, Guangzhou, China; 3 Guangdong Key Laboratory of Liver Diseases, Third Affiliated Hospital, Sun Yat-sen University, Guangzhou, China; 4 Methodist Hospital Research Institute, Weill Cornell School of Medicine, Houston, TX 77030 USA; 5 BGI Genomics, BGI-Shenzhen, Shenzhen, 518083 China; 6 Center for Reproductive Medicine, First Affiliated Hospital, Sun Yat-sen University, Guangzhou, China; 7 MRC Biostatistics Unit, Cambridge, United Kingdom; 8 Chinese Academy of Sciences Key Laboratory of Computational Biology, Max Planck Independent Research Group on Population Genomics, CAS-MPG Partner Institute for Computational Biology, Shanghai Institute for Biological Sciences, CAS, Shanghai, China; 9 Department of Orthopaedic Surgery, Sun Yat-Sen Memorial Hospital, Sun Yat-sen University, Guangzhou, China; 10 Department of Endocrinology, Fudan Institute of Metabolic Disease, Zhongshan Hospital, Fudan University, Shanghai, China; 11 Department of Endocrinology, First Affiliated Hospital, Sun Yat-sen University, Guangzhou, China; 12 Department of Prenatal Diagnostic Center, Guangzhou Women and Children’s Medical Centre, Guangzhou Medical University, Guangzhou, China; 13 Department of Pediatrics, First Af f iliated Hospital, Sun Yat-sen University, Guangzhou, China; 14 Department of Dermatology and Venereology, Third Af f iliated Hospital, Sun Yat-sen University, Guangzhou, China; 15 Department of Cardiology, Third Affiliated Hospital, Sun Yat-Sen University, Guangzhou, China; 16 Department of Dermatology, Guangdong Academy of Medical Sciences, Guangdong General Hospital, Guangzhou, China; 17 Center for Fetal Medicine, Department of Obstetrics and Gynecology, First Affiliated Hospital, Sun Yat-sen University, Guangzhou, China; 18 Guanghua School of Stomatology, Hospital of Stomatology, Guangdong Provincial Key Laboratory of Stomatology, Sun Yat-sen University, Guangzhou, China; 19 Center for Prenatal Diagnosis, Sixth Affiliated Hospital, Guangzhou Medical University, Qingyuan, China; 20 Department of Medical Genetics, Center for Genome Research, Zhongshan School of Medicine, Sun Yat-sen University, Guangzhou, China; 21 Centre for Genomic Sciences, The University of Hong Kong, Pokfulam, Hong Kong; 22 Department of Psychiatry, The University of Hong Kong, Pokfulam, Hong Kong; 23 State Key Laboratory for Cognitive and Brain Sciences, The University of Hong Kong, Pokfulam, Hong Kong; 24 Centre for Reproduction, Development and Growth, The University of Hong Kong, Pokfulam, Hong Kong; 25 Department of Pharmacology, Xinhua College, Sun Yat-sen University, Guangzhou, China; 26 School of Clinical Medicine, University of Cambridge, Cambridge, United Kingdom

## Abstract

*SLC10A1* codes for the sodium-taurocholate cotransporting polypeptide (NTCP), which is a hepatocellular transporter for bile acids (BAs) and the receptor for hepatitis B and D viruses. NTCP is also a target of multiple drugs. We aimed to evaluate the medical consequences of the loss of function mutation p.Ser267Phe in *SLC10A1*. We identified eight individuals with homozygous p.Ser267Phe mutation in *SLC10A1* and followed up for 8–90 months. We compared their total serum BAs and 6 species of BAs with 170 wild-type and 107 heterozygous healthy individuals. We performed in-depth medical examinations and exome sequencing in the homozygous individuals. All homozygous individuals had persistent hypercholanemia (*P* = 5.8 × 10^–29^). Exome sequencing excluded the involvement of other BA metabolism-associated genes in the hypercholanemia. Although asymptomatic, all individuals had low vitamin D levels. Of six adults that were subjected to bone mineral density analysis, three presented with osteoporosis/osteopenia. Sex hormones and blood lipids were deviated in all subjects. Homozygosity of p.Ser267Phe in *SLC10A1* is associated with asymptomatic hypercholanemia. Individuals with homozygous p.Ser267Phe in *SLC10A1* are prone to vitamin D deficiency, deviated sex hormones and blood lipids. Surveillance of these parameters may also be needed in patients treated with drugs targeting NTCP.

## Introduction

Bile acids (BAs) are synthesized from, and thereby regulate the levels of, cholesterol, which is the parent molecule of steroid hormones including sex hormones and adrenocortical hormones. BAs are essential for the absorption of lipids and lipid-soluble nutrients from the intestine^1^.

Sodium-taurocholate cotransporting polypeptide (NTCP), which is encoded by the *SLC10A1* gene (solute carrier family 10 member 1; GenBank accession no. 6554), transports conjugated BAs and, to a less extent, unconjugated BAs into hepatocytes from the plasma. NTCP has recently been identified as a hepatocellular receptor for hepatitis B virus (HBV) and hepatitis D virus (HDV)^2^. It also transports multiple drugs into hepatocytes, where they are metabolized^3^. Hence, NTCP is a target, or one of the targets, for several FDA-approved drugs^3^, including ezetimibe^4^ (for lowering blood lipids) and fusidic acid^5^ (antibacterial infection) used in Europe and Australia, as well as drugs that are currently in clinical trials, such as myrcludex B (anti-HBV/HDV infection)^6^.

It has been known that mutations in at least 10 genes involved in BA metabolism can cause serious diseases associated with hypercholanemia (such as progressive intrahepatic cholestasis) (Online Mendelian Inheritance in Man, OMIM http://www.ncbi.nlm.nih.gov/omim and literature^7^). However, whether mutations in *SLC10A1* cause any clear phenotype has been a riddle^8, 9^. To date there are only four cases with deleterious mutations in *SLC10A1* have been reported. One paper reported a patient with conjugated hypercholanemia, hypotonia, growth retardation, and cognitive deficiency, who was homozygous for a p.Arg252His mutation in *SLC10A1* ^8^. However, because the genetic analysis in this case was restricted to *SLC10A1*, the causal gene for the patient’s phenotypes is still open for investigation. The p.Ser267Phe mutation in *SLC10A1* (rs2296651) is one of the most prevalent deleterious variations in East Asia among genes that are involved in BA metabolism, with an allele frequency of 8–12% in individuals in Southern China (http://browser.1000genomes.org/Homo_sapiens/Variation/Population?db=core;r=14:70244693–70245693;v=rs2296651;vdb=variation;vf=1673765). This mutation has also been identified in African and Latino populations (http://exac.broadinstitute.org/variant/14-70245193-G-A). *In vitro* experiments have shown that the p.Ser267Phe mutation loses most of its function of bile acid uptake and the ability to support HBV or HDV infection^10–12^. Another paper^13^ reported a pediatric patient who was homozygous for a p.Ser267Phe mutation in *SLC10A1* and had increased blood BA levels. However, this case had complications of liver disease and dermatitis, and the infant was delivered by cesarean section due to entanglement of the umbilical cord. These complications make it difficult to interpret the medical consequence of the mutation p.Ser267Phe in *SLC10A1*. Very recently, the fourth single case was reported, in whom the heterozygous mutations of p.Ser267Phe and the truncating mutation p.Ser206Profs*12 in *SLC10A1* were identified. Apart from hypercholanemia, the clinical condition of this case raised the question whether NTCP deficiency is linked to premature loss of fetus^14^.

To unravel the clinical phenotypes and to investigate the medical consequences of the p.Ser267Phe mutation in *SLC10A1*, we have identified eight individuals with homozygous p.Ser267Phe mutation in *SLC10A1*. We performed in-depth medical investigations and exome sequencing in the homozygous individuals, and compared their BA levels with 170 wild-type and 107 heterozygous healthy individuals. Here we report our findings and their clinical implications.

## Materials and Methods

### Identification of individuals with homozygous p.Ser267Phe mutation in *SLC10A1* and recruitment of the cohort

**Figure 1:**
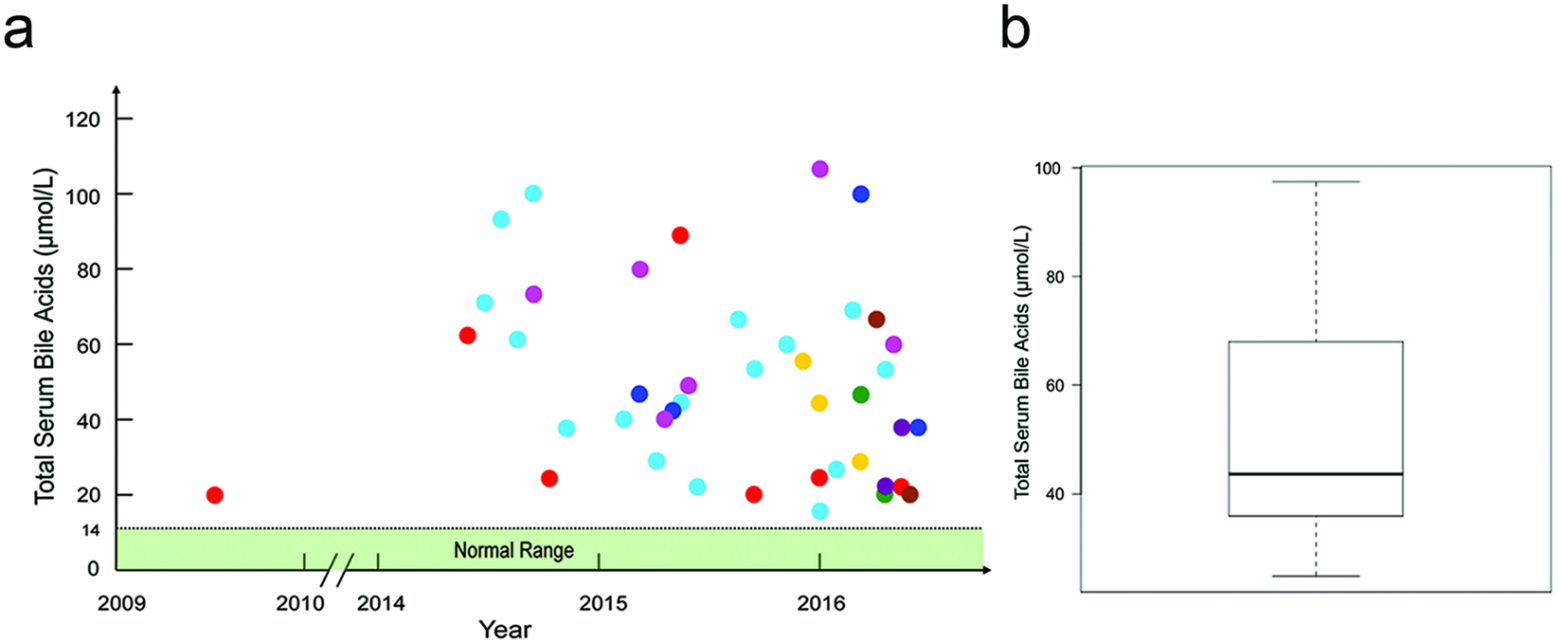
Levels of total serum bile acids (tsBAs) tested at the hospital lab and the Kingmed Diagnostics lab. (**a**) Scatter plots of tsBAs among the eight individuals who had a homozygous p.Ser267Phe genotype in *SLC10A1* tested at the hospital lab. All tests were performed on serum from blood taken first thing in the morning after at least 8 hours of fasting. Red dot: individual 1, light blue: individual 2, dark blue: individual 3, light purple: individual 4, yellow: individual 5, dark green: individual 6, brown: individual 7, and dark purple: individual 8. The light green area shows the normal range for total serum bile acids (0–14 μmol/L) according to the hospital laboratory standards. (**b**) Boxplot of tsBAs among the eight individuals who had a homozygous p.Ser267Phe genotype in *SLC10A1* according to the Kingmed Diagnostics Lab measurements. The boxplot shows the median, range, and inter-quartile range of tsBAs levels across these individuals.

During routine employer-sponsored health assessments, in which testing for total serum BAs (tsBAs) was included, two individuals (individuals 1 and 2) had higher levels of tsBAs than the hospital normal range in 2009 and 2014 respectively (Fig. 1a). The two individuals were asymptomatic. Our detailed medical investigations excluded all the causes of hypercholanemia that were known at the time, including viral hepatitis, obstruction of biliary tract, toxic factor, medication factor, autoimmune liver disease, alcoholic liver disease, fatty liver, liver cirrhosis, liver fibrosis and gastrointestinal diseases. As *SLC10A1* was a gene for which we did not know whether mutations cause any phenotype, we sequenced the gene and found that both individuals were homozygous p.Ser267Phe mutations. To further investigate the medical significance of this mutation, we started to recruit individuals who are homozygous p.Ser267Phe in *SLC10A1* from the Third Affiliate Hospital, Sun Yat-sen University, Southern China, in 2014. Our criteria were that individuals must be homozygous p.Ser267Phe in *SLC10A1* and have excluded all the known conditions that may cause increased blood tsBAs; must not bear any other predicted deleterious mutations by SIFT (http://sift.jcvi.org/) among variations reported in the 1000 Genome Project (http://browser.1000genomes.org/Homo_sapiens/Search/Results?site=ensembl&q=SLC10A1) and literature^15, 16^ (Supplementary Table S1) on *SLC10A1*; must have normal renal and liver function; must be unrelated to any other individuals in the cohort; and must not be on NTCP-targeting or interacting drugs (consecutive samples, June 2014–April 2016). We also recruited healthy and unrelated individuals who were either heterozygous or wild-type for p.Ser267Phe and did not bear any other deleterious mutations in *SLC10A1* as mentioned for the homozygous individuals from the Physical Checks Department (consecutive, February 2015–April 2016 for heterozygous and January 2016–April 2016 for wild-type individuals). In total we recruited 285 individuals, including 8 individuals who were homozygous for the p.Ser67Phe mutation (Table 1), 107 healthy heterozygous individuals, and 170 healthy wild-type individuals (Supplementary Table S2). tsBAs were measured in all the individuals. We performed in-depth medical examinations of the homozygous individuals, traced all their available medical records, and followed them up for 8–90 months (Fig. 1a, Table 1).

**Table 1:** Clinical and Demographic Data of the Individuals Who Are Homozygous for p.Ser267Phe in *SLC10A1*.

	Individual 1	Individual 2	Individual 3	Individual 4	Individual 5	Individual 6	Individual 7	Individual 8
Sex	Male	Female	Male	Male	Female	Male	Female	Female
Age (years)	48	41	64	7	48	39	29	22
Menopausal age	N/A*	N/A	N/A	N/A	46	N/A	N/A	N/A
Occupation	Medical Specialist	Medical Specialist	Farmer	Student	Cleaner	Businessman	Nurse	Medical Student
Body mass index	26.2	20.0	23.4	14.5 (−0.9 SD)^§^	20.1	25.9	17.4	19.7
Follow up (months)	90	30.5	21	25	13	10	8	8
Number of children	1	1	4	N/A	2	1	1	N/A

^*^N/A: not applicable.

^§^The normal body mass index range according to Chinese standards for individual 4 (7 years and 9 months old) is 13.0–20.6^23^.

Written informed consent was obtained from all participants. The study was approved by the Ethical Committee for Human Study, Sun Yat-sen University, and the Principles in the Declaration of Helsinki were followed.

### Clinical laboratory investigations

Serum from the homozygous, heterozygous, and wild-type individuals was collected in the morning after at least 8 hours of fasting. The initial and follow-up tsBAs measurements were conducted in the hospital laboratory by the enzyme circle method using a Total Bile Acids Assay kit (Maccure, Chengdu, China) on a Hitachi 7180 automatic biochemical analyzer (Hitachi, Ibaraki, Japan). The tsBAs and six BA species were re-tested by the independent KingMed Diagnostics Lab (http://www.kingmed.com.cn) on a separate blood sample (individual 6 had three separate blood collections and tests) using the enzyme circle method and a Total Bile Acid Reagent Kit (Dongou, Wenzhou, China) on a Roche P800 automatic biochemical analyzer (Roche, Mannheim, Germany). Liquid chromatography-tandem mass spectrometry analysis was used to measure six species of BAs—taurodeoxycholic acid (TDCA), glycodeoxycholic acid (GDCA), cholic acid (CA), deoxycholic acid (DCA), chenodeoxycholic acid (CDCA), and ursodeoxycholic acid (UDCA) on a Triple Quad 5500 mass spectrometry (AB Sciex, California, USA) using the respective reagents (Sigma-Aldrich, Missouri, USA and C/D/N Isotopes Inc, Pointe-Claire, Canada) as previously reported^17^. Blood lipids, sex and adrenocortical hormones, and vitamin A and D levels of the homozygous individuals were measured by the hospital laboratory or the KingMed Diagnostics Lab (Supplementary Tables S3 and 4). Bone density was measured by dual energy X-ray absorptiometry (DXA, Discovery DXA System, Hologic Inc, Bedford, USA).

### Sequencing and genotyping

Sanger sequencing was performed to genotype p.Ser267Phe in DNA extracted from blood. We also used Sanger sequencing to exclude all other mutations in *SLC10A1* that are predicted to be deleterious by SIFT among the Chinese in the 1000 Genome Project and in two recent reports^15, 16^ in the homozygous, heterozygous, and wild-type individuals in DNA extracted from blood (Supplementary Table S1). We also used Sanger sequencing to genotype the available parents of individuals 4 and 8. To exclude mosaicism, p.Ser267Phe was also sequenced from buccal samples of the homozygous individuals by Sanger sequencing.

To exclude possible contribution by other BA metabolism-associated genes to the hyperchalonemia, exome sequencing was performed on the Illumina and Proton platforms in the eight homozygous individuals (NCBI accession codes SRA438551; Supplementary Tables S5–7, text page 7–10, Supplementary Materials). We selected all of the genes known to cause hypercholanemia and genes coding for BA transporters in enterohepatic circulation in OMIM and in the literature^7^ (18 genes, Supplementary Table S8), and 48 other genes involved in BA metabolism in OMIM and in the literature^1, 7, 18^ (Supplementary Table S8). We retrieved and compared all the variations in the 66 genes with minor allele frequencies lower than 30% among Eastern Asian/Chinese (http://browser.1000genomes.org/Homo_sapiens) in the eight homozygous individuals (Supplementary Table S8).

To examine whether there is population stratification in the cohort (170 wild-type individuals, 107 p.Ser267Phe heterozygous individuals, and 8 p.Ser267Phe homozygous individuals), we genotyped 21 ancestry-informative markers (AIMs) in all samples in the three groups as previously reported (Supplementary Table S9)^19^ and designed a statistical test based on random sampling and using the strategy as we previously reported (text page 10–11, Supplementary Materials)^20, 21^.

### Statistical analyses

We performed separate linear regressions for each of the six BA species and tsBAs as dependent variables and on the p.Ser267Phe genotypes as an independent variable using the R software package (http://www.r-project.org) on values obtained from the KingMed Diagnostics Lab. For all statistical regression analyses we normalized each of the bile acids using a log-transform, since the raw data for each was left-skewed. For each of the BA phenotypes we explored regressions on p.Ser267Phe under both recessive and additive models. All regressions were adjusted for age and gender. For the single individual (individual 6) with three measurements of each bile acid, we used their average values in the regressions. Statistical significance was ascribed according to a Bonferroni *P*-value threshold, which was adjusted for the total number of BA phenotypes that were tested and for the use of two genetic models (i.e., our threshold for declaring significance was *P* = 0.05/14 = 0.0036). The effect estimates from our linear regressions are interpreted as the mean change in log-bile acid associated with an extra copy of the p.Ser267Phe mutation under additive models, or with the homozygous genotype under recessive models. Under both models, a negative effect indicates the mutation is associated with a reduction in the bile acid, and a positive effect is associated with an increase in tsBAs or BA species.

### Data availability statement

The raw data of the Illumina and Proton sequence reads of the whole exome sequence in the 8 individuals who are homozygous p.Ser267Phe in *SLC10A1* have been deposited in the NCBI Sequence Read Archive, accession codes SRA438551.

## Results

### The homozygous p.Ser267Phe mutation in *SLC10A1* is associated with hypercholanemia

All tsBAs tests that were performed in the hospital laboratory showed consistent hypercholanemia in all homozygous individuals (Fig. 1a). Moreover, all heterozygous and wild-type individuals were normocholanemic. The hospital measurement was confirmed by the analyses of the KingMed Diagnostics Lab, where tsBAs and 6 species of BAs were also measured in another blood collection (Table 2, Supplementary Table S2). The median of tsBAs was 3.63 times the maximal reference of the KingMed Diagnostics Lab (Fig. 1b). Under both models, the recessive and additive genetic models, tsBAs, and two species of conjugated BAs—TDCA and GDCA—were significantly elevated (Under recessive genetic model *P* = 5.8 × 10^−29^, 2.4 × 10^−14^, 2.9 × 10^−8^, respectively; Under additive genetic model *P* = 1.0 × 10^−25^, 2.6 × 10^−11^, 1.1 × 10^−8^, respectively; Table 2). One species of unconjugated BAs—CA—was also moderately increased (Under recessive genetic model *P* = 4.2 × 10^−4^; Under additive genetic model *P* = 1.4 × 10^−5^; Table 2). Analysis using the additive model also showed a moderate increase in the unconjugated DCA (*P* = 0.0013, Table 2).

**Table 2:** Statistical analyses of total serum bile acids (tsBAs) and six BA species for association with p.Ser267Phe genotype in *SLC10A1* ^#^.

	Recessive genetic model^%^			Additive genetic model		
	Effect^§^	95% CI	*P-*Value	Effect	95% CI	*P-*Value
tsBAs	2.84	(2.39, 3.28)	5.8 × 10^−29^	0.84	(0.70, 0.99)	1.0 × 10^−25^
TDCA	2.56	(1.93, 3.18)	2.4 × 10^−14^	0.71	(0.51, 0.91)	2.6 × 10^−11^
GDCA	2.87	(1.88, 3.86)	2.9 × 10^−8^	0.92	(0.61, 1.23)	1.1 × 10^−8^
CA	1.60	(0.72, 2.48)	4.2 × 10^−4^	0.61	(0.34, 0.89)	1.4 × 10^−5^
DCA	1.10	(0.17, 2.04)	0.021	0.48	(0.19, 0.77)	0.0013
CDCA	0.72	(−0.09, 1.53)	0.083	0.20	(−0.06, 0.45)	0.13
UDCA	0.55	(−0.33, 1.43)	0.22	0.00	(−0.28, 0.27)	0.99

^#^tsBAs: total serum bile acids. TDCA: taurodeoxycholic acid. GDCA: glycodeoxycholic acid. CA: cholic acid. DCA: deoxycholic acid. CDCA: chenodeoxycholic acid. UDCA: ursodeoxycholic acid. CI: confidence interval.

The *P*-values were obtained from separate linear regressions for each log-bile acid (BA) per genotype and for the total serum BAs (tsBAs), adjusted for age and gender. Our threshold for declaring significance is *P* = 0.05/14 = 0.0036.

^%^Recessive genetic model: for these linear regressions, the wild-type and heterozygous individuals were pooled and compared to the homozygous individuals.

^§^Mean change in log-bile acid associated with having the homozygous mutation under the recessive models, or with each extra mutation under the additive models. Under both models a negative effect indicates the mutation is associated with a reduction in the bile acid, and a positive effect is an increase in tsBAs or BA species.

Our Sequenom assay revealed no population stratification among the homozygous, heterozygous, and wild-type individuals, and the 21 ancestral informative markers were within the Hardy-Weinberg equilibrium (Fig. S1, Supplementary Table S9 and text page 10–11, Supplementary Materials).

These results demonstrated that homozygosity for p.Ser267Phe was associated with hypercholanemia made up of conjugated BAs, including TDCA, GDCA, and to a less extent, unconjugated CA.

### Exome sequencing excluded the involvement of other BA metabolism-associated genes

Our exome sequencing detected a total of 23 variations in the 66 genes involved in BA metabolism in the homozygous individuals, besides p.Ser267Phe in *SLC10A1* (Supplementary Table S8). Among the 23 variations, 5 were predicted to be deleterious by SIFT. Individual 1 carried the heterozygous mutation p.Val174Ala in *SLCO1B1 (solute carrier organic anion transporter family member 1B1)*; individual 4, 5, and 8 carried a heterozygous mutation p.Arg918His in *MYO5B (myosin VB)* (Supplementary Table S8); individual 1 and 3 carried a heterozygous mutation p.Val186Phe in *AMACR (alpha-methylacyl-CoA racemase)*; individual 7 had a heterozygous mutation p.Arg35Trp in *ACOX2 (acyl-CoA oxidase 2)*; and individual 8 had a heterozygous mutation p.Val61Phe in *FAH (fumarylacetoacetate hydrolase)* (Supplementary Table S8). However, as the five genes are recessive, these heterozygous variations are unlikely to be the cause of their hypercholanemia. In contrast, each and all of the hypercholanemic individuals were homozygous for p.Ser267Phe in *SLC10A1* (Supplementary Table S8). This indicates that homozygosity of p.Ser267Phe in *SLC10A1* is the likely cause of their hypercholanemia.

Sanger sequencing of buccal swabs produced the same results in p.Ser267Phe of *SLC10A1* as the blood samples in all homozygous individuals. The available parents of individuals 4 and 8 were heterozygous p.Ser267Phe carriers. These results imply that homozygous p.Ser267Phe mutation likely originated in the germline and not through mosaicism.

### The homozygous p.Ser267Phe mutation in *SLC10A1* is associated with low vitamin D levels and differences in steroid hormones and blood lipids

All of the homozygous individuals were asymptomatic. Our in-depth medical examinations showed that all of the individuals who were homozygous for the p.Ser267Phe mutation had normal liver and renal function and normal bilirubin levels. Their medical history showed that they did not have jaundice, pruritus, liver disease, or any of the conditions known to cause hypercholanemia. However vitamin D levels were reduced in all individuals relative to the preferred range (Table 3)^22^. Osteoporosis or osteopenia was diagnosed in 3 of the 6 adults who consented to testing. But none had rickets, osteomalacia, a fracture, or back or joint pain. Owing to difficulties in assessing bone density in children, we did not classify individual 4, the 7-year-old boy, into any category (Table 4). Various sex hormones in the homozygous individuals were deviated when compared with the Chinese normal range, but all homozygous adults had well-developed secondary sexual characteristics and were fertile (Table 1 and Supplementary Table S10). Various blood lipids also deviated from the normal range (Supplementary Table S11), and 24-hour urinary free cortisol levels were increased in individuals 1 and 7 (Table 3). Levels of vitamin A and aldosterone, and bleeding and coagulation times were normal in all individuals (Table 3, Supplementary Table S12). And other biochemistry and blood cell analysis were normal in all homozygous individuals (Supplementary Table S13).

**Table 3:** Vitamin D and adrenocortical hormones (cortisol and aldosterone) in individuals who are homozygous for p.Ser267Phe in *SLC10A1* ^#^.

	Individual 1	Individual 2	Individual 3	Individual 4	Individual 5	Individual 6	Individual 7	Individual 8
*25-hydroxyvitamin D* ^§^ (preferred range: 75.00–150.00) (nmol/L)								
	***54.00***	***28.00/30.00/25.00***	76.00/***56.00***	***44.00/63.00/46.00***	***48.00***	***42.00***	***53.00***	***40.00***
*Cortisol* (normal range: 118.60–618.00) (at 8:00 am) (nmol/L)								
	208.22	424.31	495.20/384.25	264.37	213.10	392.63	184.11	259.67
24-hour Urinary Free Cortisol*(normal range: 3–8y^**†**^, 1.40–20.00; ≥18 y^**†**^, 3.50–45.00) (μg/24 h)								
	***46.40***	22.30	19.70	14.90	20.10	15.50	***77.00***	15.40
*Aldosterone* (normal range: 40.00–310.00) (orthostatic) (pg/mL)								
	140.56	169.74	150.33	187.08	178.18	192.49	109.70	163.21

^#^Forward slash (/) distinguishes tests that were separately performed.

^§^Figures that deviate from the preferred range are in bold italics and are underlined.

^*^Figures that deviate from the normal range are in bold italics and are underlined.

^†^y: years.

**Table 4:** Bone mineral density of individuals who are homozygous for p.Ser267Phe in *SLC10A1* ^#,§^.

	Individual 1	Individual 2	Individual 3	Individual 4	Individual 5	Individual 6	Individual 7	Individual 8
*Lumbar vertebra* (g/cm^2^)	Did not consent	1.20 (L1–4)	***0.85*** (***L2-3***)	0.57 (L1-4)	***0.79*** (***L2-4***)	***0.75*** (***L1-4***)	0.97 (L1-4)	0.92 (L1-4)
T-Score/Z-Score		1.3/1.5	***−2.3/−1.6***	N/A^**†**^/0.4	***−2.6/−2.0***	***−3.1/−3.0***	−0.7/−0.7	−1.2/−1.0
*Femoral neck* (g/cm^2^)	Did not consent	0.78	***0.70***	0.54	***0.66***	***0.61***	0.88	0.77
T-Score/Z-Score		−0.6/−0.3	***−1.7/−0.7***	N/A/−1.4	***−1.7/−1.1***	***−2.3/−1.9***	0.3/0.3	−0.7/−0.7
*Total hip* (g/cm^2^)	Did not consent	0.90	***0.86***	0.62	***0.74***	***0.79***	0.96	0.91
T-Score /Z-Score		−0.4/−0.2	***−1.2/−0.7***	N/A/−0.7	***−1.7/−1.3***	***−1.6/−1.4***	0.2/0.2	−0.3/−0.3
*Diagnosis*		Normal	***Osteopenia***	***Unclassified***	***Osteoporosis***	***Osteoporosis***	Normal	Normal

^#^Figures that deviate from the normal range are in bold italics and are underlined.

^§^Dual energy X-ray absorptiometry (DXA) measurements were made on a Discovery DXA System (Hologic Inc, Bedford, USA). The WHO criteria for classification in adults: Male age ≥ 50 or post-menopausal phase women: Normal: T-scroe ≥ –1.0, Osteopenia: –2.5 < T-scroe < –1.0; Osteoporosis: T-scroe ≤ –2.5. Male Age < 50 or non-menopause women: Normal: Z-score > –2.0, Osteoporosis: Z-score ≤ −2.0.

^**†**^N/A: not applicable.

## Discussion

We have identified a new type of hypercholanemia that is associated with homozygosity for the p.Ser267Phe mutation in *SLC10A1*. This type of hypercholanemia is different from the previously recognized hypercholanemia in that it is associated with a different gene, and the individuals were all asymptomatic. It is different from the single reported case of the homozygous p.Arg252His mutation in *SLC10A1*, in whom neurological and developmental delay were present^8^, besides conjugated hypercholanemia. The germline origin of the mutation and the age range of our homozygous group suggest that this type of hypercholanemia is likely to begin early in life. The allele frequency of this mutation varies in different populations, with the highest incidence occurring in Southern China (8% and 12% in Chinese Han and Dai respectively) and Vietnam (11%) (http://browser.1000genomes.org/Homo_sapiens/Variation/Population?db=core;r=14:70244693–70245693;v=rs2296651;vdb=variation;vf=1673765). Taking these observations together, we propose that this “hidden” hypercholanemia affects 0.64% of the Southern Han, 1.44% of the Dai Chinese population, and 1.21% of the Vietnamese population.

The most notable finding in the homozygous individuals was the increase in conjugated and unconjugated serum BA levels. We suggest that this finding is most likely due to reduced BA transport from the portal circulation into hepatocytes. This supports the hypothesis that the physiological function of the enterohepatic circulation is not only to recycle BAs but also to clear BAs from the circulation to achieve homeostasis^9^. Alternatively, the liver may synthesize increased levels of BAs to compensate for the reduced enterohepatic recirculation in the homozygous carriers. As NTCP also transports unconjugated BAs, the increase of the unconjugated BA species observed in our study is not surprising.

BAs have detergent properties and excessive concentrations of them are thought to be toxic to the liver and other tissues, including vascular endothelium^23^. However, the results of our in-depth medical investigations seem to challenge this view, at least for vasculature at the observed blood BA levels, as our homozygous individuals all exhibited elevated tsBAs levels but had no liver and vascular damage or other apparent tissue damages.

Another consistent finding in our study cohort was the reduced vitamin D level in homozygous individuals. We observed reduced vitamin D levels in all homozygous individuals and osteoporosis or osteopenia in 3 of 6 adult homozygous individuals that were tested, which is consistent with findings from the single case study of the individual with the homozygous p.Arg252His mutation^8^ and the individual with homozygous p.Ser267Phe mutation^13^. These results suggest that the homozygous carriers are prone to vitamin D deficiency. Its level and bone density should be monitored, as interventions may be necessary to mitigate any potential effects in the homozygous individuals. This may also be appropriate for patients who undergo long-term NTCP-targeted therapies. All homozygous individuals were asymptomatic and had excluded gastrointestinal diseases, there was no evidence to support that low vitamin D level are the consequence of intestinal malabsorption. The p.Ser267Phe mutation loses most of its function of bile acid uptake in *in vitro* experiments ^10, 11^. Hypercholanemia may lead to lower level of bile acids in the intestine in all homozygous individuals. As bile acids play roles in emulsification and absorption of fat and fat-soluble vitamins, insufficient bile acids in the intestine could lead to fat-soluble vitamin D deficiency in these homozygous individuals. And studies on the related mechanisms are underway.

The deviations of levels of sex hormones, blood lipids, and urinary free cortisol seemed to show no consistent patterns in the homozygous individuals. This may be due to an adaptive response to chronic hypercholanemia, wherein the expression levels of multiple genes involved in metabolism of BAs, blood lipids and steroid hormones are altered. Whether these deviations are specifically associated with this novel form of hypercholanemia described here, and whether they are clinically significant, will require further investigation and longer follow-up. Overall results from our study demonstrate a remarkable genomic resilience and seemed reassuring for the new NTCP-targeting drugs^6^ that show strong potential for the treatment of hepatitis B and D.

The limitation of this study is the sample size. As the homozygous individuals are asymptomatic, it has taken considerable effort to recruit eight individuals.

In this study we report the identification of a new type of hypercholanemia. And our research about homozygous p.Ser267Phe individuals has implications for personalized medicine. The results from our study suggest that *SLC10A1* mutations should be screened for in individuals with asymptomatic hypercholanemia. We also recommend that levels of vitamin D be monitored or supplemented, while bone density and sex hormones, cortisol, and blood lipids should be carefully monitored in carriers who are homozygous p.Ser267Phe of *SLC10A1* and in patients who are undergoing long-term NTCP-targeted therapies, including the drugs that are currently in clinical trials, such as myrcludex B (anti-HBV/HDV infection)^6^.

## Electronic supplementary material

Supplementary material
