# Supplementary material for: Homozygous p.Ser267Phe in SLC10A1 is associated with a new type of hypercholanemia and implications for personalized medicine
Source: Sci Rep. 2017 Aug 23;7:9214. doi: 10.1038/s41598-017-07012-2 (PMC5569087; doi:10.1038/s41598-017-07012-2)
Supplement: Supplementary file 1 — Supplementary material [file 41598_2017_7012_MOESM1_ESM.doc]

**Supplementary Material**

Supplement to：**Homozygous p.Ser267Phe in *SLC10A1* is associated with a new type of hypercholanemia and implications for personalized medicine**

Ruihong Liu, B.Sc.^1, †^; Chuming Chen, M.D.^2,3, †^; Xuefeng Xia, M.D.,Ph.D.^4, †^; Qijun Liao, M.Sc.^5, †^; Qiong Wang, M.D.,Ph.D.^6^; Paul J Newcombe, Ph.D.^7^; Shuhua Xu, Ph.D.^8^; Minghui Chen, M.D.,Ph.D.^6^; Yue Ding, M.D.^9^; Xiaoying Li, M.D.,Ph.D.^10^; Zhihong Liao, M.D.,Ph.D.^11^; Fucheng Li, B.Sc.^1^; Minlian Du, M.D.^12^; Huaiqiu Huang, M.D.,Ph.D.^13^; Ruimin Dong M.D.,Ph.D.^14^; Weiping Deng, M.D.,Ph.D^15^; Ye Wang, M.D., Ph.D. ^1,16^; Binghui Zeng, M.D.^1,17^; Qihao Pan, M.D.^1,18^; Danhua Jiang, B.Sc.^1^; Hao Zeng, M.D.^1^; Pak Sham, Ph.D.,MRCPsych^19-22^; Yingnan Cao, Ph.D.^23^; Patrick H Maxwell, MBBS,DPhil^24^; Zhi-liang Gao, M.D.,Ph.D.^2,3,*^; Liang Peng, M.D.,Ph.D.^2,3,*^; Yiming Wang, M.D.,Ph.D.^25,26,5,1,*^

1. Department of Medical Genetics, Center for Genome Research, Zhongshan School of Medicine, Sun Yat-sen University, Guangzhou, China;

2. Department of Infectious Diseases, Third Affiliated Hospital, Sun Yat-sen University, Guangzhou, China;

3. Guangdong Key Laboratory of Liver Diseases, Third Affiliated Hospital, Sun Yat-sen University, Guangzhou, China;

4. Methodist Hospital Research Institute, Weill Cornell School of Medicine, Houston, TX 77030, USA;

5. BGI Genomics, BGI-Shenzhen, Shenzhen 518083, China;

6. Center for Reproductive Medicine, First Affiliated Hospital, Sun Yat-sen University, Guangzhou, China;

7. MRC Biostatistics Unit, Cambridge, United Kingdom;

8. Chinese Academy of Sciences Key Laboratory of Computational Biology, Max Planck Independent Research Group on Population Genomics, CAS-MPG Partner Institute for Computational Biology, Shanghai Institute for Biological Sciences, CAS, Shanghai, China;

9. Department of Orthopaedic Surgery, Sun Yat-Sen Memorial Hospital, Sun Yat-sen University, Guangzhou, China;

10. Department of Endocrinology, Fudan Institute of Metabolic Disease, Zhongshan Hospital, Fudan University, Shanghai, China;

11. Department of Endocrinology, First Affiliated Hospital, Sun Yat-sen University, Guangzhou, China;

12. Department of Pediatrics, First Afﬁliated Hospital, Sun Yat-sen University, Guangzhou, China;

13. Department of Dermatology and Venereology, Third Affiliated Hospital, Sun Yat-sen University, Guangzhou, China;

14. Department of Cardiology, Third Affiliated Hospital, Sun Yat-Sen University, Guangzhou, China;

15. Department of Dermatology, Guangdong Academy of Medical Sciences, Guangdong General Hospital, Guangzhou, China;

16. Center for Fetal Medicine, Department of Obstetrics and Gynecology, First Affiliated Hospital, Sun Yat-sen University, Guangzhou, China;

17. Guanghua School of Stomatology, Hospital of Stomatology, Guangdong Provincial Key Laboratory of Stomatology, Sun Yat-sen University, Guangzhou, China;

18. Center for Prenatal Diagnosis, Sixth Affiliated Hospital, Guangzhou Medical University, Qingyuan, China;

19. Centre for Genomic Sciences, the University of Hong Kong, Pokfulam, Hong Kong;

20. Department of Psychiatry, the University of Hong Kong, Pokfulam, Hong Kong;

21. State Key Laboratory for Cognitive and Brain Sciences, the University of Hong Kong, Pokfulam, Hong Kong;

22. Centre for Reproduction, Development and Growth, the University of Hong Kong, Pokfulam, Hong Kong;

23. Department of Pharmacology, Xinhua College, Sun Yat-sen University, Guangzhou, China;

24. School of Clinical Medicine, University of Cambridge, Cambridge, United Kingdom;

25. Xinhua College, Sun Yat-sen University, Guangzhou, China;

26. 5th affiliated hospital, Sun Yat-sen University, China;

^†^These authors contributed equally to this work.

***Correspondence:** Yiming Wang, Xinhua College, Sun Yat-sen University, 19 Long Dong Mei Hua Road, Tianhe District, Guangzhou, Guangdong, 510520, P.R.China. E-mail: [ywzhong@hotmail.com](mailto:ywzhong@hotmail.com), Tel: 86 20 87332055, FAX: 86 20 87331872; or Liang Peng, Department of Infectious Diseases, Third Affiliated Hospital, Sun Yat-sen University, Guangzhou, P.R.China. E-mail: [pzp33@hotmail.com](mailto:pzp33@hotmail.com), Tel: 86 20 85252372; or Zhi-liang Gao, Department of Infectious Diseases, Third Affiliated Hospital, Sun Yat-sen University, Guangzhou, P.R.China. E-mail: [zhilianggao@21cn.com](mailto:zhilianggao@21cn.com), Tel: 86 20 85252373;

**Contents**

**Text**…….....................................................................................................................Page 7-11

Exome sequencing..................................................................................................Page 7-10

Analyses for population stratification in the cohort……………............................Page 10-11

**Supplementary Tables**..............................................................................................Page 12-42

Table S1: Deleterious variations in *SLC10A1* predicted by SIFT among all variations in the gene in Chinese by the 1000 Genome Project and two recent papers....................................................................................................................Page 12

Table S2: Total serum bile acids (tsBAs) and six BA species in p.Ser267Phe genotype in *SLC10A1* ..............................................................................................................Page 13-14

Table S3: Tests performed in the hospital lab.........................................................Page 15-18

Table S4: Tests performed at the independent KingMed Diagnostics Lab.........................................................................................................................Page 19-20

Table S5: Alignment data of the 6 samples sequenced on the Illumina HiSeq platform..................................................................................................................Page 21

Table S6: Statistical data of the two samples sequenced on the Ion Torrent platform..................................................................................................................Page 22

Table S7: Alignment data of the two samples sequenced on the Ion Torrent platform..................................................................................................................Page 23

Table S8: Variations in 66 genes involved in bile acids metabolism detected by Whole Exome Sequencing in the eight individuals with hypercholanemia..................................................................................................Page 24-32

Table S9: The genotype call rate and Hardy-Weinberg Equilibrium test *P* values for the 21 ancestral informative SNPs genotyped by sequenom mass srray......................................................................................................................Page 33-34

Table S10: Sex hormones of the individuals who are homozygous for p.Ser267Phe in *SLC10A1*............................................................................................................Page 35-39

Table S11: Blood lipids of the individuals who are homozygous for p.Ser267Phe in *SLC10A1*…..........................................................................................................Page 40-41

Table S12: Vitamin A in individuals who are homozygous for p.Ser267Phe in *SLC10A1*…...........................................................................................................Page 42

Table S13: Other detailed medical examinations of the eight individuals who are homozygous of p.Ser267Phe in *SLC10A1*............................................................Page 43

Supplementary Figures.........................................................................................Page 48-49

Figure S1: Population differentiation fixation index test between individuals who are homzygous, heterozygous or wild-type p.Ser267Phe in *SLC10A1*...............................................................................................................Page 48-49

Supplementary References..................................................................................Page 50-51

**Text**

***Exome sequencing***

Samples of the 2 individuals (individuals 3 and 4) were sequenced on the Illumina HiSeq 2000 and four samples (individuals 5, 6, 7 and 8) on the Illumina HiSeq 4000 platform and two individuals (individuals 1 and 2) on the Ion Torrent Next-Generation sequencer.

*Sure Select Exome capture and Hiseq sequencing*

Sample shotgun libraries were captured for exome enrichment using Agilent Sure Select version 4 or version 5 (Agilent Technologies, Inc., Santa Clara, CA) according to the manufacturer’s protocols. Briefly, for each sample, genomic DNA was randomly fragmented by Covaris and the size distribution of the resulting DNA fragments had a peak of 150-200bp. Adapters were then ligated to both ends of the fragments. The adaptor-ligated templates were purified using Agencourt AMPure SPRI beads, and fragments with insert size ~250 bp were excised. Extracted DNA was amplified by ligation-mediated PCR, purified and hybridized to the SureSelect Biotinylated RNA library (BAITS) for enrichment. Hybridized fragments bound to the strepavidin beads, whereas non-hybridized fragments were washed out after 24h. Captured ligation-mediated PCR products were subjected to an Agilent 2100 Bioanalyzer to estimate the magnitude of enrichment. Two enriched libraries captured by Agilent Sure Select version 4 were sequenced on the Illumina HiSeq 2000 platform using paired-end 90-bases runs. The other four enriched libraries captured by Agilent Sure Select version 5 were sequenced on an Illumina HiSeq 4000 with read lengths of 100 bp.

*Read mapping and variant detection for Hiseq sequenced samples*

Sequencing reads in each individual were aligned to GRCh37.p5 using Burrows-Wheeler Aligner (BWA) software (v0.75).^1^ Aligned reads with duplicate start positions were removed using Picard (https://broadinstitute.github.io/picard). Table S5 shows statistics about alignment and coverage related to the target regions of the 6 samples sequenced by Hiseq platform. The average fold of coverage was greater than 60×, and 20× exome coverage was greater than 90% in all samples, indicating good quality. We then performed local realignment and base quality recalibration using GATK IndelRealigner and GATK BaseRecalibrator sequentially.^2^

Single nucleotide polymorphism (SNP) and small insertions/deletions (indels) detection and genotyping were performed using the GATK HaplotypeCaller tool^2^ on the targeted exome regions (We had extended 100bp for target region at both end). The variant results were filtered using GATK VariantFiltration tool with parameters “QD < 2.0 || FS > 60.0 || MQ < 40.0 || MQRankSum < -12.5 || ReadPosRankSum < -8.0”. In addition, we removed variants with Phred-like genotype quality < 20 or coverage depth < 6×. Retained high-confidence variants were annotated using the Consensus Coding Sequences Database (20140522) at the National Center for Biotechnology Information.

*Ion TargetSeq exome enrichment and Proton Sequencing*

The target regions in each of the samples was amplified using approximately 100 ng of genomic DNA on the Ion AmpliSeq Exome RDY plates and the Ion AmpliSeq HiFi Mix, generating amplicons at 240 to 280 bp. We then treated the amplicons with FuPa Reagent to partially digest the primers and phosphorylate the amplicons. The amplicons were then ligated to Proton adapters and purified to construct sequencing library. Libraries were quantified by qPCR and then sequenced on Ion Proton platform. Raw sequencing data files were processed by the Torrent sequence generation algorithm, BaseCaller, for base calling with default parameters and the sequences of each library are generated as about 160 bp single-end reads.

*Proton data analysis with Torrent Suite software*

In the Torrent Suite Software, low-quality bases were removed from the output by 1) trimming low-quality 3’ ends of the reads; 2) filtering out the entire low quality reads, including removal of short reads, removal of adapter dimmers, removal of reads lacking sequencing key, removal of reads with off-scale signal and removal of polyclonal reads. After removing low-quality bases (Table S6 shows the quality data for the two samples), the sequencing reads were aligned to GRCh37.p5 by the Torrent Mapping Alignment Program (TMAP) (http://github.com/nh13/TMAP). Table S7 shows statistics about alignment and coverage related to the target regions. The observed depths of the two samples after alignment were 123× and 141×. The 20× exome coverage was 93.56% and 95.57% respectively. Table S6 and S7 show that the data were of good quality. We then detected Single nucleotide variants (SNVs) and small Insertion/Deletions (InDels) using the Torrent Suite Variant Caller (TVC) Plugin (http://github.com/iontorrent/Torrent-Variant-Caller-stable), which is tuned to Ion Torrent data and considers quality scores and manufacturer-specific details. Retained high-confidence variants were annotated using the Consensus Coding Sequences Database (20140522) at the National Center for Biotechnology Information.

***Analyses for population stratification in the cohort***

*Sequenom Genotyping of the 21 ancestral informative markers and Hardy-Weinberg Equilibrium test*

21 ancestral informative single nucleotide polymorphisms (AIMs) were genotyped in the entire study cohort (the homozygous, heterozygous and the wild-type individuals) using Sequenom Mass Array as previously reported^3^ (Table S9). Samples with more than 2 SNPs failed in genotyping were removed. The call rate of the 21 SNPs range from 98.57% to 100% (Table S9). The exact test for Hardy-Weinberg Equilibrium (HWE) showed that no SNPs were departed from HWE (Table S9, *P* > 0.05/21 = 0.0024, Bonferroni correction). The genotype was successful in 279 samples including 8 individuals who are homozygous for p.Ser267Phe in *SLC10A1*, 104 individuals who are heterozygous and 167 wild-type individuals.

*Evaluation of population stratification among the cohort using ancestral informative markers*

To examine whether there is population stratification in the cohort, we designed a statistical test based on random sampling and using the strategy as we previously reported.^4,5^ To reduce the effect of differences in the sample sizes among the three groups, an identical number (n = 8) of individuals to the homozygous group were randomly sampled from the wild-type group and compared to the remaining samples of the group by calculating genetic difference (F_ST_)^6^ for 21 AIMs. This random sampling procedure was repeated 10,000 times so that a distribution of F_ST_ was created. This distribution was further normalized (Figure S1, upper panel) to perform a standard statistical test based on normal distribution. The same analysis was also performed to compare the homozygous and heterozygous groups (Figure S1, lower panel).

**Supplementary Tables**

**Table S1. Deleterious variations in *SLC10A1* predicted by SIFT* among all variations in the gene in Chinese by the 1000 Genome Project and two recent papers^7,8^**

| **Nucleotide Change** | **Amino acid Change** | **rs** |
| --- | --- | --- |
| c.263T>C | p.Ile88Thr | rs148467625 |
| c.357‐1G>A |  | rs748759248 |
| c.402G>A | p.Met134Ile | rs771742051 |
| c.515T>C | p.Ile172Thr | rs756182169 |
| c.538C>T | p.Arg180Trp |  |
| c.547T>C | p.Tyr183His |  |
| c.553C>T | p.Arg185Cys | rs200149939 |
| c.557A>G | p.Tyr186Cys |  |
| c.565A>T | p.Lys189Stp |  |
| c.598G>A | p.Val200Met | rs202213974 |
| c.665A>G | p.Leu222Ser |  |
| c.767T>C | p.Met256Thr | rs750857596 |
| c.800C>T | p.Ser267Phe | rs2296651 |
| c.836T>C | p.Ile279Thr | rs72547507 |

*Predicted by SIFT (http://sift.jcvi.org).

**Table S2. Total serum bile acids (tsBAs) and six BA species in p.Ser267Phe genotype in *SLC10A1*^#, §^**

| **Bile acid species**  Normal range | | **tsBAs (μmol/L)** | | | | **TDCA (μmol/L)** | | | | | **GDCA (μmol/L)** | | | | **CA (μmol/L)** | | | | | | | **DCA (μmol/L)** | | | | **CDCA (μmol/L)** | | | | | | | | **UDCA (μmol/L)** | |  |
| --- | --- | --- | --- | --- | --- | --- | --- | --- | --- | --- | --- | --- | --- | --- | --- | --- | --- | --- | --- | --- | --- | --- | --- | --- | --- | --- | --- | --- | --- | --- | --- | --- | --- | --- | --- | --- |
|  |  | 0–12 | | | | 0–0.26 | | | | | 0–1.31 | | | | | | | 0.02–2.04 | | | | 0–1.63 | | | | 0.02–2.89 | | | | | | | | 0–0.80 | |  |
| **Homozygote individuals** | | | | | | | | | | | | | | | | | | | | | | | | | | | | | | | | | | | |  |
| Individual 1 | | 97.40 | | | | | | 0.67 | | 7.93 | | | | 0.22 | | | | | | 0.80 | | | | | 0.64 | | | | 0.05 | | | | | | | |
| Individual 2 | | 62.90 | | | | | | 0.33 | | 2.78 | | | | 1.17 | | | | | | 0.41 | | | | | 1.78 | | | | 1.39 | | | | | | | |
| Individual 3 | | 37.60 | | | | | | 0.51 | | 4.73 | | | | 3.66 | | | | | | 1.35 | | | | | 1.28 | | | | 0.12 | | | | | | | |
| Individual 4 | | 38.70 | | | | | | 0.32 | | 2.79 | | | | 0.51 | | | | | | 0.53 | | | | | 0.56 | | | | 0.47 | | | | | | | |
| Individual 5 | | 34.20 | | | | | | 0.58 | | 6.00 | | | | 0.27 | | | | | | 0.83 | | | | | 0.77 | | | | 0.41 | | | | | | | |
| Individual 6 | | | 48.50/22.20/58.90 | | | | <0.012/<0.012/<0.012 | | | | | 0.020/0.014/<0.012 | | | | | 1.59/0.24/2.55 | | | | <0.012/<0.012/0.039 | | | | | | 3.37/0.65/2.83 | | | | | | | | 0.58/0.30/0.48 |  |
| Individual 7 | | 73.10 | | | | | | 1.68 | | 11.12 | | | | 0.20 | | | | | | 1.95 | | | | | 0.26 | | | | | 0.03 | | | | | | |
| Individual 8 | | 24.80 | | | | | | 0.32 | | 3.51 | | | | 0.07 | | | | | | 0.45 | | | | | 0.04 | | | | | | | | 0.02 | | | |
| **Heterozygote individuals** *(Mean±SD)* *(N = 107, Female:male: 71:36, Age (mean±SD): 31.66±9.57)* | | | | | | | | | | | | | | | | | | | | | | | | | | | |  | | | | | | | |  |
|  | | 4.50±2.52 | | | 0.06±0.06 | | | | | 0.41±0.38 | | | 0.26±0.42 | | | | | | 0.43±0.34 | | | | | 0.46±0.52 | | | | | | | 0.15±0.18 | | | | |  |
| **Wide-type individuals** *(Mean±SD)* *(N = 170, Female:male: 126:44, Age (mean±SD ): 38.79±10.73)* | | | | | | | | | | | | | | | | | | | | | | | | | | | |  | | | | | | | |  |
|  | 2.71±1.60 | | | 0.05±0.05 | | | | | 0.27±0.29 | | | | | | | 0.22±0.37 | | | | 0.33±0.32 | | | 0.49±0.54 | | | | | | | | | 0.23±0.32 | | | |  |

^#^tsBAs: total serum bile acids. TDCA: taurodeoxycholic acid. GDCA: glycodeoxycholic acid. CA: cholic acid. DCA: deoxycholic acid. CDCA: chenodeoxycholic acid. UDCA: ursodeoxycholic acid.

^§^Forward slash (/) distinguishes tests that were separately performed.

**Table S3. Tests performed in the hospital lab**

| **Test** | **Equipment** | **Reagent** | **Method** |
| --- | --- | --- | --- |
| 25-hydroxyvitamin D | Multiskan MK3 (Thermo scientific, Vantaa, Finland) | OCTEIA 25-Hydroxy Vitamin D kit  (Immunodiagnostic Systems Limited, Wear, UK ) | Euzyme linked immunosorbent assay |
| Cortisol (serum) | ADVIA Centaur XP  (Siemens Healthcare Diagnostics Inc, Massachusetts, USA) | Cortisol Kit  (Siemens Healthcare Diagnostics Inc, Massachusetts, USA) | Direct chemical luminescence |
| Aldosterone | Autolumo A2000 chemiluminescence immunoassay  (AutoBio, Zhengzhou, China) | ALD CLIA Microparticles  (AutoBio, Zhengzhou, China) | Microparticles Chemical luminescence method |
| Follicle Stimulating Hormone | ADVIA Centaur XP Immunoassay System  (Siemens Healthcare Diagnostics Inc, Massachusetts, USA) | Follicle Stimulating Hormone kit  (Siemens Healthcare Diagnostics Inc, Massachusetts, USA) | Direct chemical luminescence |
| Prolactin | ADVIA Centaur XP Immunoassay System  (Siemens Healthcare Diagnostics Inc, Massachusetts, USA) | ADVIA Centaur Prolactin kit  (Siemens Healthcare Diagnostics Inc, Massachusetts, USA) | Direct chemical luminescence |
| Progesterone | ADVIA Centaur XP Immunoassay System  (Siemens Healthcare Diagnostics Inc, Massachusetts, USA) | ADVIA Centaur Progesterone kit  (Siemens Healthcare Diagnostics Inc, Massachusetts, USA) | Direct chemical luminescence |
| Luteinizing Hormone | ADVIA Centaur XP Immunoassay System  (Siemens Healthcare Diagnostics Inc, Massachusetts, USA) | Luteinizing Hormone kit  (Siemens Healthcare Diagnostics Inc, Massachusetts, USA) | Direct chemical luminescence |
| Estradiol | ADVIA Centaur XP Immunoassay System  (Siemens Healthcare Diagnostics Inc, Massachusetts, USA) | ADVIA Centaur Estradiol kit  (Siemens Healthcare Diagnostics Inc, Massachusetts, USA) | Direct chemical luminescence |
| Androstenedione | Immulite 2000 Automated chemiluminescence immunoassay analyzer  (Siemens Healthcare Diagnostics Products Limited, UK) | IMMULITE2000 Androstenedione Kit  (Siemens Healthcare Diagnostics Products Limited, Gwynedd, UK) | Chemical luminescence |
| Dehydroepiandrosterone sulfate | ARCHITECT I2000 Immunoassay Analyzer  (Abbott, Illinois,USA) | ARCHITECT DHEA-S Reagent Kit  (Abbott GmbH & Co. KG, Barcelona, Spain) | Delayed one-step immunoassay |
| Sex hormone binding globulin | ARCHITECT I2000 Immunoassay Analyzer  (Abbott, Illinois, USA) | ARCHITECT SHBG Reagent Kit  (Abbott GmbH & Co. KG, Barcelona, Spain ) | Two-step immunoassay |
| Total testosterone | ADVIA Centaur XP Immunoassay System  (Siemens Healthcare Diagnostics Inc, Massachusetts, USA) | ADVIA Centaur Testosterone kit  (Siemens Healthcare Diagnostics Inc, Massachusetts, USA) | Direct chemical luminescence |
| Total Cholesterol | Hitachi 7180 automatic biochemical analyzer (Hitachi, Ibaraki, Japan) | Total Cholesterol Assay Kit  (Maccure, Chengdu, China) | COD-CE-PAP Method |
| Triglyceride | Hitachi 7180 automatic biochemical analyzer (Hitachi, Ibaraki, Japan) | Triglyceride Assay Kit  (Maccure, Chengdu, China) | GOP-PAP Method |
| HDL cholesterol | Hitachi 7180 automatic biochemical analyzer (Hitachi, Ibaraki, Japan) | Homogenous method  (Sekisui Medical co., Ltd, Ibaraki, Japan) | Homogenous method |
| LDL cholesterol | Hitachi 7180 automatic biochemical analyzer (Hitachi, Ibaraki, Japan) | Homogenous method  (Sekisui Medical co., Ltd, Ibaraki, Japan) | Homogenous method |
| Apolipoprotein A1 | Hitachi 7180 automatic biochemical analyzer (Hitachi, Ibaraki, Japan) | Apolipoprotein A1 & B Assay Kit  (Maccure, Chengdu, China) | Immunoturbidimetry Method |
| Apolipoprotein B | Hitachi 7180 automatic biochemical analyzer (Hitachi, Ibaraki, Japan) | Apolipoprotein A1 & B Assay Kit  (Maccure, Chengdu, China) | Immunoturbidimetry Method |
| Lipoprotein a | Hitachi 7180 automatic biochemical analyzer (Hitachi, Ibaraki, Japan) | Lipoprotein a Assay Kit  (Maccure, Chengdu, China) | Latex Enhanced Immunoturbidimetry Method |

**Table S4. Tests performed at the independent KingMed Diagnostics lab**

| **Test** | **Equipment** | **Reagent** | | **Method** |
| --- | --- | --- | --- | --- |
| Vitamin A | 1289 Infinity ultra performance  liquid chromatograph  (Agilent, California, USA) | Standards (RECIPE, Germany)  Other reagents  (Sigma – Aldrich, Missouri, USA) | | Ultra performance liquid chromatograph |
| 24 hour Urinary Free Cortisol | API4000plus Massspectrometry  (AB, USA) | Standards (DR, Germany and TRC, Canada; )  Other reagents  (Merk-ChemicalsKGaA, Germany) | | Liquid chromatography–tandem mass spectrometry |
| 17 α-hydroxyprogesterone | GC-2016 radiation immunity arithmometer  (Zhongjia, Hefei, China) | OHP-CT Kit  (CIS, France) | | Radioimmunoassay |
| Total testosterone | Agilent G6495 mass spectroscopy  （Agilent, California, USA） | Standards (Sigma, USA)  Other reagents  (Merk-ChemicalsKGaA, Germany) | | Liquid chromatography–tandem mass spectrometry |
| Free testosterone | GC-2016 radioimmunoassay counter  (Zhongjia, Hefei, China) | Free TESTOSTERONE -RIA-CT Kit  (DIAsource ImmunoAssays SA, Louvain-la-Neuve, Belgium) | Radioimmunoassay | |

**Table S5. Alignment data of the 6 samples s**equenced on the Illumina HiSeq platform

|  | **Individual 3*** | **Individual 4*** | **Individual 5**^§^ | **Individual 6** ^§^ | **Individual 7**^§^ | **Individual 8** ^§^ |
| --- | --- | --- | --- | --- | --- | --- |
| Mapping rate on genome (%) | 99.50 | 99.57 | 99.04 | 98.47 | 98.80 | 98.66 |
| Average sequencing depth on target | 88.82 | 77.03 | 63.99 | 86.40 | 101.26 | 81.21 |
| Fraction of target covered ≥ 1× (%) | 99.70 | 99.20 | 99.64 | 99.84 | 99.80 | 99.78 |
| Fraction of target covered ≥ 4× (%) | 99.20 | 98.40 | 99.11 | 99.54 | 98.60 | 99.51 |
| Fraction of target covered ≥10× (%) | 97.50 | 97.00 | 97.44 | 98.72 | 98.92 | 98.65 |
| Fraction of target covered ≥ 20× (%) | 93.20 | 93.50 | 91.43 | 96.03 | 96.31 | 95.43 |

*Individual sequenced on Hiseq 2000 platform.

^§^Individual sequenced on Hiseq 4000 platform.

**Table S6. Statistical data of the two samples sequenced o**n the Ion Torrent platform

|  | **Individual 1** | **Individual 2** |
| --- | --- | --- |
| Total number of reads (M) | 44.04 | 50.55 |
| Total number of bases (G) | 7.774 | 8.915 |
| Mean length of reads | 176 | 176 |
| Peak length of reads | 226 | 226 |
| Percent of base with Q17+ | 87.22% | 86.98% |
| Percent of base with Q20+ | 81.12% | 81.15% |

**Table S7. Alignment data of the two samples s**equenced on the Ion Torrent platform.

|  | **Individual 1** | **Individual 2** |
| --- | --- | --- |
| Number of mapped reads | 43,124,009 | 50,232,887 |
| Mapping rate on genome | 97.93% | 99.37% |
| Average sequencing depth on target | 123.0 | 141.4 |
| Uniformity of coverage* | 91.70% | 93.41% |
| Fraction of target covered ≥1 × | 99.39% | 99.35% |
| Fraction of target covered ≥20 × | 93.56% | 95.57% |
| Fraction of target covered ≥100 × | 52.25% | 61.76% |
| Target bases with no strand bias^§^ | 83.65% | 83.25% |
| Percent of base with AQ17+ ^¶^ | 92.96% | 93.67% |
| Percent of base with AQ20+ | 89.09% | 89.24% |

*The percentage of bases in the target regions covered by at least 0.2 × the average sequencing depth.

^§^The percentage of all targets that did not show a bias towards forward or reverse strand read alignments. An individual target is considered to have read bias if it has at least 10 reads and the fraction of forward or reverse reads to total reads greater than 70%.

^¶^ Percent of base with alignment quality value of 17 or higher.

**Table S8. Variations in 66 genes involved in bile acids metabolism detected by whole exome sequencing in the eight individuals with hypercholanemia^#, §^**

| **Gene** | **Variation** | **rs** | **AF**^§^ | **Inheritance pattern** | **Functional Effects**^¶^ | **Genotype of Individual 1/2/3/4/5/6/7/8** | **Shared by all** | |
| --- | --- | --- | --- | --- | --- | --- | --- | --- |
| **Variations in 18 genes that are known to cause hypercholanemia and genes code for bile acids transporters in enterohepatic circulation** | | | | | | | | |
| ***SLC10A1*** | **p.Ser267Phe**  **(c.800C>T)** | **rs2296651** | **0.081** | **?** | **Deleterious** | **Hom**^†^**/Hom/Hom/Hom/Hom/Hom/Hom/Hom** | | **Yes** |
| *SLCO1B1* | p.Val174Ala  (c.521T>C) | rs4149056 | 0.119 | DR^‡^ | Deleterious | Het^*^/WT^‖^/WT/WT/ WT/WT/WT/WT | | No |
| *MYO5B* | p.Arg918His  (c.2753G>A) | rs2298624 | 0.271 | AR | Deleterious | WT/WT/WT/Het/Het/WT/WT/Het | | No |
|  | p.Gly1321Glu  (c.3962G>A) | rs1942418 | 0.238 |  | Tolerated | Het/Het/Het/WT/WT/Hom/WT/WT | | No |
|  | p.Lys307Asn  (c. 921G>T) | rs17659179 | 0.0014 |  | Tolerated | WT/WT/WT/WT/WT/WT/WT/Het | | No |
| *ABCC2* | p.Val417Ile  (c.1249G>A) | rs2273697 | 0.110 | AR | Tolerated | WT/Het/WT/WT/Het/WT/Het/WT | | No |
| *TJP2* | p.Arg24His  (c.G71A) | rs4493966 | 0.091 | AR | Tolerated | WT/WT/WT/WT/WT/WT/WT/Het | | No |
|  | p.Gln132Lys  (c.C394A) | rs41305539 | 0.033 |  | Tolerated | WT/WT/WT/WT/WT/WT/WT/Het | | No |
| *EPHX1* | p.His139Arg  (c. 416 A>G) | rs2234922 | 0.124 | ? | Tolerated | Het/WT/Het/WT/WT/WT/Het/WT | | No |
| *ABCB11* | None | | | AR |  | WT/WT/WT/WT/WT/WT/WT/WT | | No |
| *ABCB4* | None | | | AR,AD |  | WT/WT/WT/WT/WT/WT/WT/WT | | No |
| *ATP8B1* | None | | | AR,AD |  | WT/WT/WT/WT/WT/WT/WT/WT | | No |
| *SLC10A2* | None | | | AR |  | WT/WT/WT/WT/WT/WT/WT/WT | | No |
| *SLCO1B3* | None | | | DR^‡^ |  | WT/WT/WT/WT/WT/WT/WT/WT | | No |
| *BAAT* | None | | | ? |  | WT/WT/WT/WT/WT/WT/WT/WT | | No |
| *SLC25A13* | None | | | AR |  | WT/WT/WT/WT/WT/WT/WT/WT | | No |
| *NR1H4* | None | | | AR |  | WT/WT/WT/WT/WT/WT/WT/WT | | No |
| *SLC51A* | None | | | ? |  | WT/WT/WT/WT/WT/WT/WT/WT | | No |
| *SLC51B* | None | | | ? |  | WT/WT/WT/WT/WT/WT/WT/WT | | No |
| *ABCC3* | None | | | ? |  | WT/WT/WT/WT/WT/WT/WT/WT | | No |
| *ABCC4* | None | | | ? |  | WT/WT/WT/WT/WT/WT/WT/WT | | No |
|  |  |  |  |  |  |  | |  |
| **Variations in 48 other genes involved in bile acids metabolism** | | | | | | | | |
| *AMACR* | p.Val186Phe  (c.556 G>T) | rs34677 | 0.205 | AR | Deleterious | Het/WT/Het/WT/WT/WT/WT/WT | | No |
| *ACOX2* | p.Arg35Trp  (c.103C>T) | rs142584671 | 0 | AR | Deleterious | WT/WT/WT/WT/WT/WT/Het/WT | | No |
| *NOTCH2* | p.Ile1689Phe  (c.5065A>T) | rs60854092 | 0.024 | AD | Tolerated | WT/WT/WT/WT/WT/WT/Het/WT | | No |
| *CFTR* | p.Ile556Val  (c.1666A>G) | rs75789129 | 0.067 | AR,AD | Tolerated | Het/WT/WT/WT/WT/WT/WT/WT | | No |
| *SLCO4A1* | p.Val78Ile  (c.232G>A) | rs1047099 | 0.157 | ? | Tolerated | WT/WT/Het/WT/WT/WT/WT/WT | | No |
| *ABCG5* | p.Gln604Glu  (c.1810C>G) | rs6720173 | 0.138 | AR | Tolerated | WT/WT/Het/WT/WT/WT/WT/WT | | No |
| *ABCG8* | p.Thr400Lys  (c.1199C>A) | rs4148217 | 0.105 | AR | Tolerated | WT/WT/WT/WT/Het/Het/Het/WT | | No |
| *SERPINA1* | p.Arg125His  (c.374G>A) | rs386607395 | 0.228 | AR | Tolerated | Het/WT/WT/Het/WT/WT/Het/WT | | No |
| *SLC27A5* | p.Met50Thr  (c.149T>C) | rs35350976 | 0.062 | ? | Tolerated | WT/WT/WT/WT/WT/WT/Het/WT | | No |
|  | p.Arg53Trp  (c.157C>T) | rs34415062 | 0.062 |  | Tolerated | WT/WT/WT/WT/WT/WT/Het/WT | | No |
| *FH* | p.Pro26Leu  (c. 77C>T) | rs187226800 | 0.019 | AR,AD | Tolerated | WT/WT/WT/WT/Het/WT/WT/WT | | No |
| *CYP39A1* | p.Arg23Pro  (c. 68G>C) | rs12192544 | 0.138 | ? | Tolerated | WT/Het/Het/WT/Het/WT/Het/WT | | No |
|  | p.Arg103His  (c.308G>A) | rs2277119 | 0.229 |  | Tolerated | WT/Het/Het/WT/Het/WT/Het/WT | | No |
| *FAH* | p.Val61Phe  (c.181G>T) | rs151264725 | 0.014 | AR | Deleterious | WT/WT/WT/WT/WT/WT/WT/Het | | No |
| *NPC1* | p.Arg1266Gln  (c.3797G>A) | rs1805084 | 0.186 | AR | Tolerated | WT/Het/WT/WT/WT/Het/WT/WT | | No |
| *HSD3B7* | None | |  | AR |  | WT/WT/WT/WT/WT/WT/WT/WT | | No |
| *SLC4A2* | None | |  | ? |  | WT/WT/WT/WT/WT/WT/WT/WT | | No |
| *CYP7A1* | None | |  | ? |  | WT/WT/WT/WT/WT/WT/WT/WT | | No |
| *ATP8B1* | None | |  | AR,AD |  | WT/WT/WT/WT/WT/WT/WT/WT | | No |
| *AKR1D1* | None | |  | AR |  | WT/WT/WT/WT/WT/WT/WT/WT | | No |
| *CYP7B1* | None | |  | AR |  | WT/WT/WT/WT/WT/WT/WT/WT | | No |
| *JAG1* | None | |  | AR |  | WT/WT/WT/WT/WT/WT/WT/WT | | No |
| *FABP6* | None | |  | ? |  | WT/WT/WT/WT/WT/WT/WT/WT | | No |
| *ABCD3* | None | |  | AR |  | WT/WT/WT/WT/WT/WT/WT/WT | | No |
| *VPS33B* | None | |  | AR |  | WT/WT/WT/WT/WT/WT/WT/WT | | No |
| *VIPAS39* | None | |  | AR |  | WT/WT/WT/WT/WT/WT/WT/WT | | No |
| *CYP27A1* | None | |  | AR |  | WT/WT/WT/WT/WT/WT/WT/WT | | No |
| *HSD17B4* | None | |  | AR |  | WT/WT/WT/WT/WT/WT/WT/WT | | No |
| *CLDN1* | None | |  | AR |  | WT/WT/WT/WT/WT/WT/WT/WT | | No |
| *BCS1L* | None | |  | AR |  | WT/WT/WT/WT/WT/WT/WT/WT | | No |
| *ABCC6* | None | |  | AR,AD |  | WT/WT/WT/WT/WT/WT/WT/WT | | No |
| *SLCO1A2* | None | |  | ? |  | WT/WT/WT/WT/WT/WT/WT/WT | | No |
| *CYP8B1* | None | |  | ? |  | WT/WT/WT/WT/WT/WT/WT/WT | | No |
| *SLC10A7* | None | |  | ? |  | WT/WT/WT/WT/WT/WT/WT/WT | | No |
| *SLC22A1* | None | |  | ? |  | WT/WT/WT/WT/WT/WT/WT/WT | | No |
| *SLC22A7* | None | |  | ? |  | WT/WT/WT/WT/WT/WT/WT/WT | | No |
| *ABCB1* | None | |  | ? |  | WT/WT/WT/WT/WT/WT/WT/WT | | No |
| *SLC47A1* | None | |  | ? |  | WT/WT/WT/WT/WT/WT/WT/WT | | No |
| *VDR* | None | |  | AR,AD |  | WT/WT/WT/WT/WT/WT/WT/WT | | No |
| *HNF1A* | None | |  | AR,AD |  | WT/WT/WT/WT/WT/WT/WT/WT | | No |
| *NR0B2* | None | |  | AR,AD |  | WT/WT/WT/WT/WT/WT/WT/WT | | No |
| *FGF19* | None | |  | ? |  | WT/WT/WT/WT/WT/WT/WT/WT | | No |
| *NR5A2* | None | |  | ? |  | WT/WT/WT/WT/WT/WT/WT/WT | | No |
| *NR3C1* | None | |  | AD |  | WT/WT/WT/WT/WT/WT/WT/WT | | No |
| *RARA* | None | |  | ? |  | WT/WT/WT/WT/WT/WT/WT/WT | | No |
| *PPARA* | None | |  | ? |  | WT/WT/WT/WT/WT/WT/WT/WT | | No |
| *CIRH1A* | None | |  | ？ |  | WT/WT/WT/WT/WT/WT/WT/WT | | No |
| *GALT* | None | |  | AR |  | WT/WT/WT/WT/WT/WT/WT/WT | | No |
| *LIPA* | None | |  | AR |  | WT/WT/WT/WT/WT/WT/WT/WT | | No |
| *NPC2* | None | |  | AR |  | WT/WT/WT/WT/WT/WT/WT/WT | | No |

***^#^***18 Genes that are known to cause hypercholanemia and genes code for bile acids transporters in rnterohepatic circulation and 48 genes involved in metabolism of bile acids are retrieved from OMIM (http://www.ncbi.nlm.nih.gov/omim) and literature. ^9-11^

^§^Allele frequency in Southern Han Chinese (CHS) by phase III 1000 Genome Project (http://www.1000genomes.org). Variations with allele frequency less than 0.3 among Eastern Asian/Chinese are retrieved.

^¶^Predicted by SIFT (http://sift.jcvi.org).

^†^Hom: homozygous.

^‡^DR: Digenic recessive inheritance. Rotor -type hyperbilirubinemia (HBLRR) was caused by digenic recessive inheritance of mutations in *SLCO1B1* and *SLCO1B3* genes by OMIM.

^*^Het: heterozygous.

^‖^WT: wild type.

**Table S9. The genotype call rate and Hardy-Weinberg Equilibrium test *P* values for the 21 ancestral informative SNPs genotyped by sequenom mass array**

| **rs ID** | **Chr.** | **Call rate** | **HWE*P* value**^#^ |
| --- | --- | --- | --- |
| rs10917532 | 1 | 100.00% | 0.528 |
| rs883201 | 1 | 100.00% | 0.769 |
| rs10910470 | 1 | 100.00% | 0.234 |
| rs10929660 | 2 | 100.00% | 1.000 |
| rs11128125 | 3 | 100.00% | 0.622 |
| rs17579988 | 4 | 100.00% | 0.005 |
| rs4428236 | 4 | 100.00% | 1.000 |
| rs997427 | 5 | 99.64% | 0.360 |
| rs304141 | 5 | 99.64% | 0.299 |
| rs11745587 | 5 | 100.00% | 1.000 |
| rs1558205 | 6 | 100.00% | 0.423 |
| rs10504726 | 8 | 100.00% | 0.770 |
| rs4354269 | 8 | 100.00% | 0.140 |
| rs1981500 | 9 | 100.00% | 0.278 |
| rs10858883 | 12 | 100.00% | 0.717 |
| rs10775022 | 13 | 100.00% | 0.811 |
| rs8005568 | 14 | 100.00% | 0.800 |
| rs1715919 | 15 | 100.00% | 0.631 |
| rs6117562 | 20 | 98.57% | 0.630 |
| rs8127081 | 21 | 100.00% | 1.000 |
| rs860236 | 22 | 100.00% | 1.000 |

^#^HWE: Hardy-Weinberg Equilibrium.

The threshold for declaring significance was *P* = 0.05/21 = 0.0024.

**Table S10. Sex hormones of the individuals who are homozygous for p.Ser267Phe in *SLC10A1*^#^**

|  | | | | **Individual 1** | | | | | | **Individual 2** | **Individual 3** | | | **Individual 4** | | | | | | | | | **Individual 5** | | | | | **Individual 6** | | | | | | | | **Individual 7** | | | | | **Individual 8** | | | |
| --- | --- | --- | --- | --- | --- | --- | --- | --- | --- | --- | --- | --- | --- | --- | --- | --- | --- | --- | --- | --- | --- | --- | --- | --- | --- | --- | --- | --- | --- | --- | --- | --- | --- | --- | --- | --- | --- | --- | --- | --- | --- | --- | --- | --- |
| *Follicle-Stimulating Hormone*  (normal range: male: 1.40-18.10. Female: follicular phase, 2.50-10.20; ovulatory phase, 3.40-33.40; luteal phase, 1.50-9.10; menopause, 23.00-116.30) (mIU/mL) | | | | | | | | | | | | | | | | | | | | | | | | | | | | | | | | | | | | | | | | | | | | |
|  | | | 3.29/3.18 | | | | | 3.85  (Day 1) ^¶^  ***1.20***  (Day 20) ^¶^  2.10  (Day18) ^¶^ | | | ***18.98***/16.43/***19.89*** | | | | | N/A^§^ | | | | | | | | 59.11/92.58  (menopause) | | | | | | 5.85 | | | |  | | | | | 6.25  (Day 5) ^¶^ | | | | 4.58  (Day 15) ^¶^ | |
| *Prolactin* (normal range: male: 48.00-375.00. Female: non-pregnancy, 59.00-619.00; pregnant, 206.00-4420.00; post-menopausal, 38.00-430.00) (μIU/mL) | | | | | | | | | | | | | | | | | | | | | | | | | | | | | | | | | | | | | | | | | | | | |
|  | | 88.66/97.70 | | | | | ***681.09***/595.48/488.27 (non-pregnancy) | | | | | 223.42/325.98/165.15 | | | | | | | | | | N/A | | | 131.47/121.94  (menopause) | | | | | | | 140.02 | | | 156.93 (non-pregnancy) | | | | | 224.94 (non-pregnancy) | | | | |
| *Progesterone* (normal range: male: 0.89-3.88; female: follicular phase 0.50-4.50, ovulatory phase 14.12-89.14, luteal phase 10.62-81.28, post-menopausal phase 0.00-2.32) (nmol/L) | | | | | | | | | | | | | | | | | | | | | | | | | | | | | | | | | | | | | | | | | | | | |
|  | | | | | 2.11/2.56 | | | 1.83  (Day 1) ^¶^  42.58  (Day 20) ^¶^  28.19  (Day 18) ^¶^ | | | ***0.81/0.77***/0.96 | | | | N/A | | | | | | | | | | 1.07/0.92  (menopause) | | | | | | | 1.02 | | | | | | | 2.31  (Day 5) ^¶^ | | ***2.96***  (Day 15) ^¶^ | | | |
| *Luteinizing Hormone*  (normal range: male: 1.50-9.30. Female: follicular phase, 1.90-12.50; ovulatory phase, 8.70-76.00; luteal phase, 0.50-16.90; menopause, 5.90-54.00) (mIU/mL) | | | | | | | | | | | | | | | | | | | | | | | | | | | | | | | | | | | | | | | | | | | | |
|  | | | 3.07/3.20 | | | | | | 6.03  (Day 1) ^¶^  3.56  (Day 20) ^¶^  3.35  (Day 18) ^¶^ | | ***17.53/14.75/17.55*** | | | | | | N/A | | | | | | | | 53.60***/66.67***  (menopause) | | | | | | | 3.31 | | | | | | | 5.30  (Day 5) ^¶^ | | 10.81  (Day 15) ^¶^ | | | |
| *Estradiol*  (normal range: male <146.10; female follicular phase: 71.60-529.20, ovulatory phase 234.50-1309.10, luteal phase 204.80-786.10, post-menopausal phase <118.20) (pmol/L) | | | | | | | | | | | | | | | | | | | | | | | | | | | | | | | | | | | | | | | | | | | | |
|  | ***242.08/345.53//256.55*** | | | | | | | | 122.96  (Day 1) ^¶^  773.03  (Day 20) ^¶^  540.64  (Day 18) ^¶^ | | ***189.91/187.04/203.56*** | | | | | | | | | N/A | | | | | | | ***228.59/168.27***  (menopause) | | | | | | ***192.84*** | | | | | 343.11  (Day 5) ^¶^ | | | | 752.81  (Day 15) ^¶^ | | |
| *Androstenedione* (normal range: male, 2.10-10.80; female, 1.00-11.50) (nmol/L) | | | | | | | | | | | | | | | | | | | | | | | | | | | | | | | | | | | | | | | | | | | | |
|  | | | | | | 7.91/10.80 | | ***12.40/12.60/22.50*** | | | | | 5.00/6.70 | | | | N/A | | | | | | | | 10.20 | | | | ***16.30*** | | | | | | | | | | 10.70 | | ***19.40*** | | | |
| *17 α-hydroxyprogesterone* (normal range: adult male: 0.31-2.17. Female: follicular phase, 0.10-0.80; luteal phase, 0.27-2.90) (ng/mL) | | | | | | | | | | | | | | | | | | | | | | | | | | | | | | | | | | | | | | | | | | | | |
|  | | | | | | 0.54 | | 0.49  (Day 1) ^¶^ | | | 1.21 | | | | | | | N/A | | | | | | 0.36  (menopause) | | | | | | | | 0.93 | | | | | 0.43  (Day 5) ^¶^ | | | | | | 0.69  (Day 15) ^¶^ | |
| *Dehydroepiandrosterone sulfate*  (normal range^&^: male: 35-44y, 3.80-13.10; 45-54y, 3.70-12.10; 55-64y, 1.30-9.80. Female: 20-24y, 3.60-11.10; 25-34y, 2.00-11.10; 35-44y, 3.80-13.10; 45-54y, 1.50-7.70) (μmol/L) | | | | | | | | | | | | | | | | | | | | | | | | | | | | | | | | | | | | | | | | | | | | |
|  | | | | | | ***14.27*** | | 4.59 | | | 3.95 | | | | | | N/A | | | | | | | | | 1.75 | | | 6.84 | | | | | | | | 5.41 | | | | | | | 10.28 |
| *Sex hormone-binding globulin* (normal range: male: 11.20-78.10; female: 11.70-137.20) (nmol/L) | | | | | | | | | | | | | | | | | | | | | | | | | | | | | | | | | | | | | | | | | | | | |
|  | | | | | | 43.90 | | 82.30 | | | 73.10 | | | | | | N/A | | | | | | | 90.50 | | | | | 26.80 | | | | | | | | 78.20 | | | | | | | 32.90 |
| *Total testosterone (direct chemical luminescence)* (normal range: male, 242.07-827.09; female, 14.41-74.93) (ng/dL） | | | | | | | | | | | | | | | | | | | | | | | | | | | | | | | | | | | | | | | | | | | | |
|  | | | | | | 451.01/354.18 | | 33.14/39.48/32.85 | | | | 710.37/619.31/586.46 | | | | | | | N/A | | | | | ***<10.09***/15.56 | | | | | | | 528.53 | | | | | | ***<10.09*** | | | | | | | ***13.26*** |
| *Total testosterone（LS-MS/MS）*^#^ (normal range: male ≥19 years old, 240.00-950.00; female ≥19 years old, 8.00-60.00) (ng/dL） | | | | | | | | | | | | | | | | | | | | | | | | | | | | | | | | | | | | | | | | | | | | |
|  | | | | | | 503.50 | | 26.60 | | | 706.60 | | | | | | | | | | N/A | | | 10.30 | | | | | 468.30 | | | | | | | | 18.90 | | | | | | | 35.90 |
| *Free testosterone* (normal range^†^: male: 20-39y, 8.90-42.50; 40-59y, 6.60-30.00; >60y, 4.90-21.60. Female: <20y, 0-3.09; 20-39y, 0-3.09; 40-59y, 0-2.60) (pg/mL) | | | | | | | | | | | | | | | | | | | | | | | | | | | | | | | | | | | | | | | | | | | | |
|  | | | | | | 11.04 | | 0.84 | | | 9.28 | | | | | | | | | | N/A | | | 0.33 | | | | | 10.59 | | | | | | | | 0.97 | | | | | | | 1.54 |

^#^Forward slash (/) distinguishes tests that were separately performed; figures that deviate are in bold italics and are underlined.

^§^ N/A: not applicable

^¶^ Day of the cycle

^#^LS-MS/MS: liquid chromatography–tandem mass spectrometry.

^†^y: years

**Table S11. Blood lipids of the individuals who are homozygous for p.Ser267Phe in *SLC10A1^#^***

|  | | | | **Individual 1** | | **Individual 2** | | **Individual 3** | **Individual 4** | | **Individual 5** | | | **Individual 6** | **Individual 7** | **Individual 8** | | |
| --- | --- | --- | --- | --- | --- | --- | --- | --- | --- | --- | --- | --- | --- | --- | --- | --- | --- | --- |
| *Total Cholesterol* (normal range: 3.10-5.70) (mmol/L) | | | | | | | | | | | | | | | | | | |
|  | 4.90/***7.62/6.34*** | | | | | 4.33/3.56 | | 5.37/4.95/4.97 | | 3.73/4.39 | | 5.47/***6.29*** | | 5.65/***6.20*** | 3.62/4.22 | ***2.27***/3.41 | | |
| *Triglyceride* (normal range: 0.34-1.92) (mmol/L) | | | | | | | | | | | | | | | | | | |
|  | | | 1.11/1.92/1.82 | | | 1.66/0.66 | | 1.66/1.28/1.01 | | 0.56/0.71 | | 0.90/0.88 | | ***2.00***/1.59 | 0.58/0.53 | | 0.46/0.60 | |
| *High-density lipoprotein cholesterol* (normal range: 0.78-2.00) (mmol/L) | | | | | | | | | | | | | | | | | | |
|  | | | 1.19/1.43/1.44 | | | 1.55/1.43 | | 0.98/1.07/1.09 | | 1.40/1.58 | | 1.48/***2.06*** | | 1.88/1.73 | 1.71/1.96 | | 0.98/1.37 | |
| *Low-density lipoprotein cholesterol* (normal range: 2.07-3.10) (mmol/L) | | | | | | | | | | | | | | | | | | |
|  | | | ***3.38/5.21/4.47*** | | | 2.33/***2.05*** | | ***3.70/3.73/3.50*** | | ***1.97***/2.29 | | ***3.50/4.07*** | | ***3.23/3.92*** | ***1.79***/2.11 | | ***1.23/1.98*** | |
| *Apolipoprotein A1* (normal range: 1.00-1.60) (g/L) | | | | | | | | | | | | | | | | | | |
|  | | 1.41/1.53/1.55 | | | | ***1.66***/1.46 | | 1.48/1.42/1.52 | | 1.31/1.56 | | ***1.67/1.63*** | | ***1.67*** | ***1.68*** | | | 1.36 |
| *Apolipoprotein B* (normal range: 0.60-1.10) (g/L) | | | | | | | | | | | | | | | | | | |
|  | | 1.01/***1.71/1.50*** | | | | 1.06/***0.47*** | | ***1.34/1.28***/1.07 | | 0.60/0.62 | | ***1.20/1.11*** | | ***1.40*** | 0.60 | | | ***0.56*** |
| *Lipoprotein (a)* (normal range: 0.00-300.00) (mg/L) | | | | | | | | | | | | | | | | | | |
|  | | | 28.00/38.00/16.00 | | 69.00/83.00 | | ***328.00/365.00/345.00*** | | | 171.00/153.00 | | | 150.00/125.00 | ***789.00*** | 214.00 | | | 112.00 |

***^#^***Forward slash (/) distinguishes tests that were separately performed; deviated figures are in bold italics and are underlined.

**Table S12. Vitamin A in individuals who are homozygous for p.Ser267Phe in *SLC10A1***

|  | **Individual 1** | **Individual 2** | **Individual 3** | **Individual 4** | | **Individual 5** | **Individual 6** | **Individual 7** | **Individual 8** |
| --- | --- | --- | --- | --- | --- | --- | --- | --- | --- |
| *Vitamin A* (normal range: > 0.20 ) (mg/L) | | | | | | | | | |
|  | 0.77 | 0.42 | 0.48 | 0.28 | 0.37 | | 1.00 | 0.27 | 0.24 |

**Table S13 Other detailed medical examinations of the eight individuals who are homozygous of p.Ser267Phe in *SLC10A1***

|  | **Individual 1** | **Individual 2** | **Individual 3** | **Individual 4** | **Individual 5** | **Individual 6** | **Individual 7** | **Individual 8** |
| --- | --- | --- | --- | --- | --- | --- | --- | --- |
| **Liver Function and Biochemistry** | | | | | | | | |
| *Aspartate aminotransferase (AST)* (normal range: 15.00-40.00 (Male) ; 13.00-35.00 (Female)) (U/L) | | | | | | | | |
|  | 26.00 | 22.00 | 23.00 | 29.00 | 27.00 | 29.00 | 30.00 | 17.00 |
| *Alanine transaminase (ALT)* (normal range: 3.00-35.00) (U/L) | | | | | | | | |
|  | 33.00 | 21.00 | 25.00 | 11.00 | 12.00 | 18.00 | 29.00 | 10.00 |
| *Albumin* (normal range: 36.00-51.00) (g/L) | | | | | | | | |
|  | 43.10 | 44.10 | 41.30 | 45.00 | 46.50 | 49.80 | 45.80 | 45.90 |
| *Globulin* (normal range: 25.00-35.00) (g/L) | | | | | | | | |
|  | 30.70 | 27.70 | 31.60 | 25.10 | 32.60 | 29.70 | 30.60 | 29.60 |
| *Total Bilirubin* (normal range: 4.00-23.90) (μmol/L) | | | | | | | | |
|  | 9.60 | 6.00 | 11.10 | 11.70 | 7.80 | 10.20 | 15.00 | 13.20 |
| *Direct Bilirubin* (normal range: 0.00-6.80) (μmol/L) | | | | | | | | |
|  | 2.00 | 2.30 | 3.60 | 3.80 | 1.50 | 2.90 | 4.60 | 4.90 |
| *γ-Glutamyl transpeptidase* (normal range: 10.00-60.00 (Male); 7.00-45.00 (Female)) (U/L) | | | | | | | | |
|  | 46.00 | 40.00 | 22.00 | 12.00 | 28.00 | 43.00 | 15.00 | 11.00 |
| *Alkaline Phosphatase* (normal range: 45.00-125.00 (Male); 35.00-135.00 (Female)) (U/L) | | | | | | | | |
|  | 62.00 | 69.00 | 101.00 | 189.00/214.00^#^ | 61.00 | 97.00 | 57.00 | 55.00 |
| *Ammonia* (normal range: 0.00-54.00) (μmol/L) | | | | | | | | |
|  | 15.10 | 11.00 | 16.50 | 20.60 | 9.30 | 11.90 | 10.60 | 15.30 |
| *Fasting blood glucose* (normal range: 3.90-6.10) (mmol/L) | | | | | | | | |
|  | 5.41 | 4.70 | 5.85 | 4.52 | 4.29 | 4.94 | 4.61 | 5.13 |
| *Abdominal ultrasound (including liver, spleen, pancreas and* [*biliary system*](http://www.baidu.com/link?url=wtoKGVVbQPeZ1JZueDE8GUaflVUo1n5HLL49XdGS8RjSYVw6LHF_b9U3mU2MSsAzI8ClvPHGDGNayuEb4dRcuDkGLToM36tvNPFei7wsmxamQmDxh2jzyCbqEafmqgTh&wd=&eqid=98103395003b8f9c00000003579df4c8)*)* | | | | | | | | |
|  | Polyps, but no obstruction. | Normal | Normal | Normal | Normal | Normal | Normal | Normal |
| **Hepatitis B Antigens and Antibodies** | | | | | | | | |
| *Hepatitis B surface antigen* | | | | | | | | |
|  | - | - | - | - | - | - | - | - |
| *Hepatitis B surface antibody* | | | | | | | | |
|  | - | + | + | + | + | + | + | + |
| *Hepatitis B e antigen* | | | | | | | | |
|  | - | - | - | - | - | - | - | - |
| *Hepatitis B e antibody* | | | | | | | | |
|  | - | - | - | - | - | - | - | - |
| *Hepatitis B core antibody* | | | | | | | | |
|  | - | - | - | - | - | - | - | - |
| **Renal Function** | | | | | | | | |
| *Urea ammonia* (normal range: 2.40-8.20) (mmol/L) | | | | | | | | |
|  | 5.85 | 3.36 | 5.15 | 5.70 | 4.22 | 4.45 | 4.41 | 2.64 |
| *Creatinine* (normal range: 31.80-116.00 (Male); 31.80-91.00 (Female) (μmol/L) | | | | | | | | |
|  | 86.00 | 56.00 | 81.00 | 38.00 | 46.90 | 92.00 | 61.00 | 55.00 |
| **Blood Cell Analysis** | | | | | | | | |
| *White Blood Count* (normal range: 3.50-9.50 (more than 6 years old); 5.0-10.0 (0-6 years old)) (×10E9/L) | | | | | | | | |
|  | 5.95 | 5.62 | 6.89 | 7.21 | 4.44 | 6.08 | 4.78 | 5.37 |
| *Red Blood Cell* (normal range: 4.30-5.80 (Male: more than 6 years old); 4.00-4.50 (Male: 0-6 years old); 3.80-5.10 (Female)) (×10E12/L) | | | | | | | | |
|  | 5.03 | 4.11 | 4.69 | 4.79 | 4.17 | 4.98 | 4.14 | 4.55 |
| *Hemoglobin* (normal range: 130-175 (Male: more than 6 years old); 120-140 (Male: 0-6 years old);115-150 (Female) (g/L) | | | | | | | | |
|  | 147 | 126 | 145 | 136 | 123 | 153 | 123 | 111 |
| *Platelets* (normal range: 100-350) (×10E9/L) | | | | | | | | |
|  | 224 | 289 | 235 | 304 | 211 | 291 | 174 | 305 |
| *Neutrophils Percentage* (normal range: 0.400-0.750 (more than 6 years old), 0.400-0.600 (0-6 years old)) | | | | | | | | |
|  | 0.485 | 0.596 | 0.645 | 0.486 | 0.522 | 0.456 | 0.550 | 0.620 |
| *Lymphocyte Percentage* (normal range: 0.200-0.500 (more than 6 years old), 0.400-0.500 (0-6 years old)) | | | | | | | | |
|  | 0.434 | 0.404 | 0.260 | 0.404 | 0.410 | 0.475 | 0.368 | 0.291 |
| *Monocyte Percentage* (normal range: 0.030-0.100) | | | | | | | | |
|  | 0.045 | 0.077 | 0.048 | 0.043 | 0.052 | 0.054 | 0.061 | 0.076 |
| *Eosnophils Percentage* (normal range: 0.004-0.080) | | | | | | | | |
|  | 0.034 | 0.021 | 0.046 | 0.061 | 0.011 | 0.008 | 0.015 | 0.009 |
| *Basophilic Granulocyte Percentage* (normal range: 0-0.010) | | | | | | | | |
|  | 0.002 | 0.002 | 0.001 | 0.006 | 0.005 | 0.007 | 0.006 | 0.004 |
| *Immaturate Granulocyte Percentage* (normal range: 0-0.010) | | | | | | | | |
|  | 0.000 | 0.002 | 0.001 | 0.000 | 0.000 | 0.003 | 0.002 | 0.004 |

^#^ The normal range for boys at the ages of 6-7 years’ old in the region is 137-294 U/L.^12^

**Supplementary Figures**


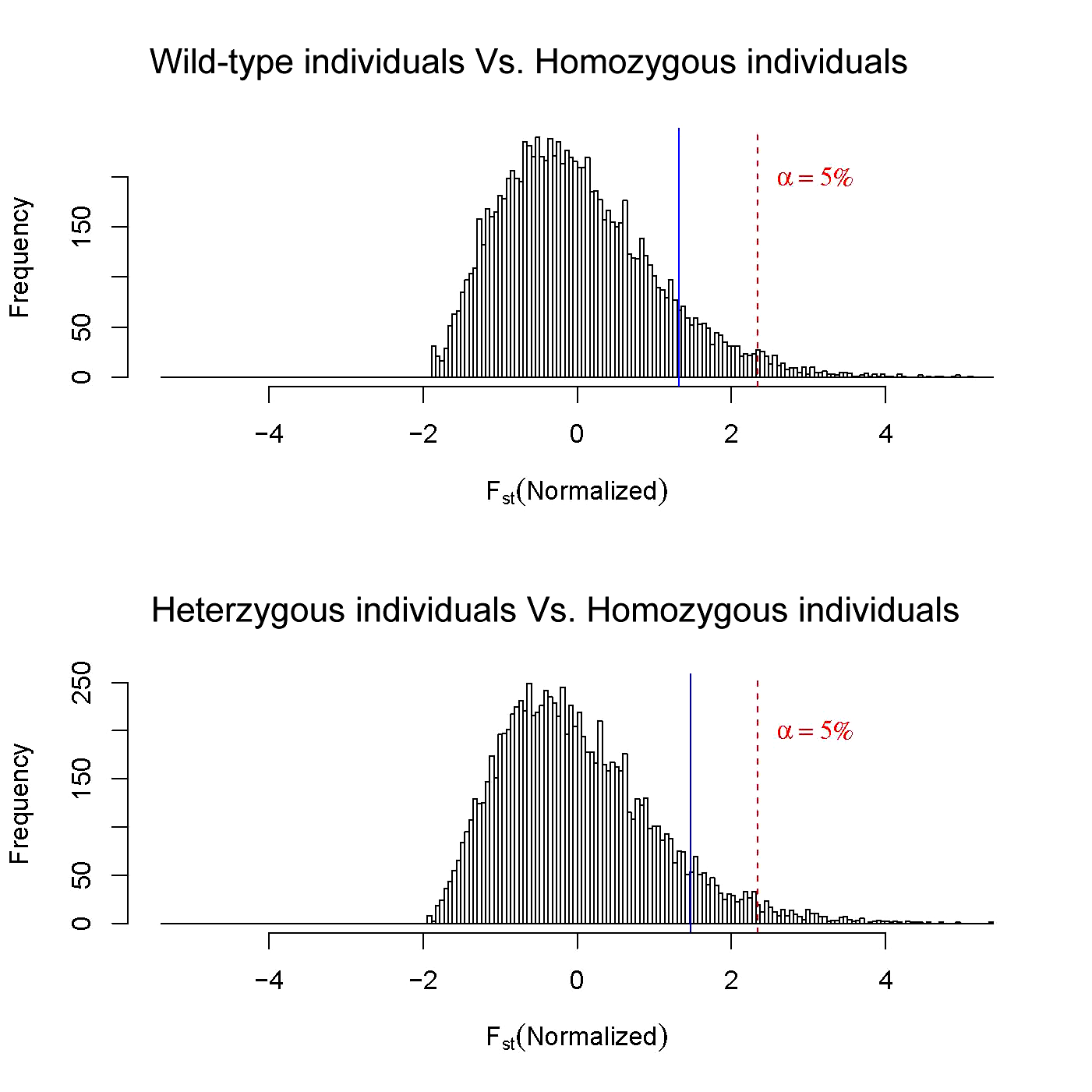


**Figure S1. Population differentiation fixation index test between individuals who are homzygous, heterozygous or wild-type p.Ser267Phe in *SLC10A1***

In the upper panel of the figure, the red dashed line indicates the significant level of 5% (assuming 5% false positive rate), the blue line represents the genetic difference between homozygous individuals and wild-type individuals (measured by F_ST_ calculated from 21 ancestral informative single nucleotide polymorphisms (AIMs)). As shown in the figure (upper panel), homozygous individuals do not show significant difference from wild-type individuals based on 21 AIMs (*P* = 0.135). The same analysis was also performed for comparing homozygous individuals and heterozygous individuals. Again, as shown in the figure (lower panel), homozygous individuals do not show significant difference from heterozygous individuals based on 21 AIMs (*P* = 0.0982). Taken together, the difference in the bile acids levels between the homozygous and heterozygous and wild-type individuals was not resulted from population stratification of the samples.

**Supplementary** **References**

1. Li, H. & Durbin, R. Fast and accurate short read alignment with Burrows-Wheeler transform. *Bioinformatics* **25,** 1754-1760 (2009).

2. DePristo, M.A. *et al.* A framework for variation discovery and genotyping using next-generation DNA sequencing data. *Nat Genet* **43,** 491-498 (2011).

3. Peng, L. *et al.* The p.Ser267Phe variant in SLC10A1 is associated with resistance to chronic hepatitis B. *Hepatology* **61,** 1251-1260 (2015).

4. Lou, H. *et al*. A 3.4-kb Copy-Number deletion near EPAS1 is significantly enriched in High-Altitude tibetans but absent from the denisovan sequence. *Am J Hum Genet* **97,** 54-66 (2015).

5. Qin, P. *et al.* A panel of ancestry informative markers to estimate and correct potential effects of population stratification in Han Chinese. *Eur J Hum Genet* **22,** 248-253 (2014).

6. Weir BS & Cockerham CC. Estimating F-Statistics for the Analysis of Population Structure. *Evolution* **38,** 1358-1370(1984).

7. Yang, J. *et al.* A genetic variant of the NTCP gene is associated with HBV infection status in a Chinese population. *BMC Cancer* **16,** 211 (2016).

8. Hu, H.H. *et al.* The rs2296651 (S267F) variant on NTCP (SLC10A1) is inversely associated with chronic hepatitis B and progression to cirrhosis and hepatocellular carcinoma in patients with chronic hepatitis B. *Gut* **65,** 1514-1521 (2016).

9. Boyer, J.L. Bile formation and secretion. *Compr Physiol* **3,** 1035-1078 (2013).

10. Alrefai, W.A. & Gill, R.K. Bile acid transporters: structure, function, regulation and pathophysiological implications. *Pharm Res* **24,** 1803-1823 (2007).

11. Qiu, Y.L. *et al.* Defects in MYO5B are associated with a spectrum of previously undiagnosed low gamma-glutamyltransferase cholestasis. *Hepatology* (2016).

12. Liao CM. *et al.* [Investigation on the reference range of serum alkaline phosphatase in children in Guangzhou City.] *Chinese Journal of Misdiagnostics* **11,** 7695-7696 (2011).
